# Using zebrafish larval models to study brain injury, locomotor and neuroinflammatory outcomes following intracerebral haemorrhage

**DOI:** 10.12688/f1000research.16473.2

**Published:** 2018-11-08

**Authors:** Siobhan Crilly, Alexandra Njegic, Sarah E. Laurie, Elisavet Fotiou, Georgina Hudson, Jack Barrington, Kirsty Webb, Helen L. Young, Andrew P. Badrock, Adam Hurlstone, Jack Rivers-Auty, Adrian R. Parry-Jones, Stuart M. Allan, Paul R. Kasher

**Affiliations:** 1 Division of Neuroscience and Experimental Psychology, School of Biological Sciences, Faculty of Biology, Medicine and Health, Manchester Academic Health Science Centre, The University of Manchester, Oxford Road, Manchester, M13 9PT, UK; 2 Division of Cardiovascular Sciences, School of Medical Sciences, Faculty of Biology, Medicine and Health, Manchester Academic Health Science Centre, The University of Manchester, Oxford Road, Manchester, M13 9PT, UK; 3 Division of Cancer Sciences, School of Medical Sciences, Faculty of Biology, Medicine and Health, Manchester Academic Health Science Centre, The University of Manchester, Oxford Road, Manchester, M13 9PT, UK; 4 Division of Evolution and Genomic Sciences, School of Biological Sciences, Faculty of Biology, Medicine and Health, Manchester Academic Health Science Centre, The University of Manchester, Oxford Road, Manchester, M13 9PT, UK

**Keywords:** Intracerebral haemorrhage, zebrafish, neuroinflammation, animal models, pre-clinical

## Abstract

Intracerebral haemorrhage (ICH) is a devastating condition with limited treatment options, and current understanding of pathophysiology is incomplete. Spontaneous cerebral bleeding is a characteristic of the human condition that has proven difficult to recapitulate in existing pre-clinical rodent models. Zebrafish larvae are frequently used as vertebrate disease models and are associated with several advantages, including high fecundity, optical translucency and non-protected status prior to 5 days post-fertilisation. Furthermore, other groups have shown that zebrafish larvae can exhibit spontaneous ICH. The aim of this study was to investigate whether such models can be utilised to study the pathological consequences of bleeding in the brain, in the context of pre-clinical ICH research.

Here, we compared existing genetic (bubblehead) and chemically inducible (atorvastatin) zebrafish larval models of spontaneous ICH and studied the subsequent disease processes. Through live, non-invasive imaging of transgenic fluorescent reporter lines and behavioural assessment we quantified brain injury, locomotor function and neuroinflammation following ICH. We show that ICH in both zebrafish larval models is comparable in timing, frequency and location. ICH results in increased brain cell death and a persistent locomotor deficit. Additionally, in haemorrhaged larvae we observed a significant increase in macrophage recruitment to the site of injury. Live *in vivo* imaging allowed us to track active macrophage-based phagocytosis of dying brain cells 24 hours after haemorrhage. Morphological analyses and quantification indicated that an increase in overall macrophage activation occurs in the haemorrhaged brain.

Our study shows that in zebrafish larvae, bleeding in the brain induces quantifiable phenotypic outcomes that mimic key features of human ICH. We hope that this methodology will enable the pre-clinical ICH community to adopt the zebrafish larval model as an alternative to rodents, supporting future high throughput drug screening and as a complementary approach to elucidating crucial mechanisms associated with ICH pathophysiology.

Research highlights **Scientific benefits:**

- No surgery is required so the models more accurately recapitulate spontaneous ICH
- Ease of genetic manipulation associated with zebrafish allows for the use of transgenic and mutant lines
- Transparency of zebrafish larvae allows for non-invasive monitoring of cellular processes over time (live imaging)

**3Rs benefits:**

- Unprotected zebrafish larvae (before 5 dpf) can be used as an alternative to rodent models in pre-clinical ICH research

**Practical benefits:**

- High fecundity of zebrafish pairings means that a large sample size of genetically comparable siblings can be produced easily
- Zebrafish are a lower cost organism to host than rodents
- Experimental timeline is less time consuming than typical rodent studies which look at long term outcomes of stroke commonly up to 3 months

**Current applications:**

- Elucidating immediate pathological outcomes of spontaneous ICH in the brain of intact animals

**Potential applications:**

- High throughput drug screens for ICH therapies
- Replacing the current rodent models for analysis of immediate ICH pathology

## Introduction

Intracerebral haemorrhage (ICH) accounts for 10–15% of strokes and has the worst stroke outcomes, with a 1-month case fatality of 40% and disability in most survivors ( An *et al*., 2017). The effects of ICH in the brain are biphasic. Primary injury following an ICH event arises due to an influx of blood into the brain and haematoma expansion which increases intracranial pressure on cerebral structures causing neuronal death and cell necrosis ( Mracsko & Veltkamp, 2014; Xi *et al*., 2006). A secondary wave of injury is induced by the breakdown of blood compounds which activates the immune system, further exacerbating cellular damage and death in the brain parenchyma and induces a breakdown in blood-brain barrier integrity ( Lok *et al*., 2011). This inflammatory component is considered a viable therapeutic target in ICH and other forms of stroke ( Veltkamp & Gill, 2016) and targeting the toxic insult of blood components after bleed onset is being investigated clinically ( Yeatts *et al*., 2013). However at present, apart from acute and chronic blood pressure lowering, we have no specific treatments to prevent ICH or improve patient outcomes once bleeding has occurred.

Despite representing a significant public health burden ( WHO, 2017), an understanding of the fundamental pathogenesis of ICH is still lacking. Pre-clinical studies to-date have depended heavily on rodent models of ICH, which have improved our knowledge of the basic mechanisms associated with the disease ( Casals *et al*., 2011). However, current rodent models of ICH involve severe surgical intervention, poorly recapitulating the spontaneous and immediate nature of the human disease and presenting welfare implications associated with severe experimental procedures in mammals ( ASPA, 1986 amendments 2012). Autologous blood injection and collagenase injection models ( Andaluz *et al*., 2002) are used worldwide, and typical experimental groups include 6-8 rats, sacrificed at various time points for *ex vivo* histological analysis, which can result in ~150 animals used per publication ( Wang *et al*., 2018) highlighting scope for a change of strategy from a 3Rs perspective. Unfortunately, this research has not yet resulted in the translation of any specific drugs to the clinic ( Kellner & Connolly, 2010; Kirkman *et al*., 2011). Potential reasons for this include difficulties in observing cellular responses in ‘real-time’ within whole brains of intact live animals, and the invasive and artificial procedures required to induce cerebral haematomas ( MacLellan *et al*., 2010; Selim *et al*., 2018). Mammalian models of spontaneous ICH models do exist, such as cerebral amyloid angiopathy co-morbidity studies and use of hypertensive mice, but their usefulness is limited due to variability in haematoma size, timing and location ( Alharbi *et al*., 2016). Alternative and complementary approaches are therefore needed to bridge the ‘translational gap’ for novel drug target discovery in ICH.

Zebrafish ( *Danio rerio*) are becoming an increasingly popular tool for studying cerebrovascular disease ( Walcott & Peterson, 2014). Due to the production of hundreds of offspring from a single adult pairing, zebrafish larvae can be utilised for high-throughput drug screening, thus offering an attractive model for pre-clinical research. Larval transparency and the availability of numerous transgenic reporter lines amount to an extremely powerful system for studying and visualising cellular responses and disease processes *in-vivo* in real time. Prior to 5 days post fertilisation (dpf), larval zebrafish are not a protected species (in the UK) and could therefore replace a significant number of protected mammals used for pre-clinical study. Furthermore, spontaneous brain-specific bleeding can be observed in zebrafish larvae using non-invasive techniques ( Eisa-Beygi *et al*., 2013; Liu *et al*., 2007), thereby eliminating specific constraints associated with mammalian models. As such, the use of larval zebrafish models of ICH could offer critical insight into the immediate cellular responses after a bleed to support the rodent community and provide a potential platform for future drug discovery addressing pre-clinical ICH priorities ( Selim *et al*., 2018).

As previously described, zebrafish larvae exposed to atorvastatin (ATV) at 24 hours post-fertilisation (hpf) exhibit spontaneous cerebral-specific blood vessel rupture at the onset of circulation (~33 hpf) ( Eisa-Beygi *et al*., 2013; Huang *et al*., 2018; Li *et al*., 2017; Shen *et al*., 2013). Comparably, the ‘bubblehead’ (bbh) mutant line, which expresses a hypomorphic mutation in the *arhgef7* gene, encoding the Rac GEF βpix, also exhibit spontaneous ICH and hydrocephalus within a similar time frame to the ATV model ( Liu *et al.*, 2007; ten Klooster *et al.*, 2006). ICH is induced through comparable mechanistic defects in both ATV and bbh models ( Eisa-Beygi & Rezaei, 2016) ( Supplementary Figure 1). Although several groups have utilised these models to study the development and integrity of the cerebrovasculature ( Buchner *et al.*, 2007; Huang *et al.*, 2018; Li *et al.*, 2017; Liu *et al.*, 2007; Yang *et al.*, 2017), they have not yet been used to study the pathological and neuroinflammatory consequences of bleeding in the zebrafish larval brain in the context of human ICH. Furthermore, drug intervention studies have focussed on preventing cerebrovascular rupture in zebrafish rather than targeting the disease outcomes, which represent a more realistic therapeutic avenue. In this study, we show that spontaneous ICH in non-protected zebrafish larvae induces quantifiable pathological and inflammatory phenotypes that relate to the human condition. As such, these data support the use of this model species as a valuable complementary system for pre-clinical ICH research.

## Methods

A detailed protocol of the experimental procedure is available in Supplementary File 1

### Zebrafish *(Danio rerio)* strains

Zebrafish were raised and maintained at The University of Manchester Biological Services Unit under standard conditions as previously described ( Westerfield, 2000). Adult zebrafish husbandry was approved by the University of Manchester Animal Welfare and Ethical Review Board. All experiments were performed in accordance with U.K. Home Office regulations (PPL:70/9091) and reported according to ARRIVE guidelines. Transgenic lines used in this study include macrophage-specific lineage *mpeg1:*mCherry (constructed in-house as previously described ( Ellett *et al.*, 2011)), neutrophil-specific *mpo:*GFP ( Renshaw *et al.*, 2006), erythroid-specific *gata1:*dsRed ( Traver *et al.*, 2003) and *ubiq:*secAnnexinV-mVenus, a reporter for cell death (re-derived in house ( Morsch *et al.*, 2015)) on wild-type, nacre ( *mitfa* ^w2/w2^ *)* and mutant ( *bbh* ^m292^) backgrounds. Fertilized embryos were collected from natural spawning and incubated at 28°C in standard E3 embryo medium and staged according to standard guidelines ( Kimmel *et al.*, 1995). At experiment end, zebrafish larvae were terminated prior to protected status using a lethal overdose of 4% MS222 anaesthesia and freezing at -20°C.

A completed ARRIVE checklist is available in Supplementary File 2

### ICH models

ICH was modelled using genetic (bbh) and chemical (ATV) approaches. For the bbh line ( Liu *et al.*, 2007), embryos were obtained from adult in-crosses from heterozygous *bbh* ^m292^ mutant animals (maintained on wild-type and transgenic reporter backgrounds). For the ATV model, nacre or transgenic embryos were dechorionated at 24 hpf and transferred to clean petri dishes in E3 embryo medium. ATV (Sigma-Aldrich, PZ0001) was solubilised in distilled water to a stock concentration of 0.5 mM. Embryos (n=100) were treated with a final concentration of 1 μM ATV through water bath incubation at 28°C for 24 hours and equivalent numbers were left as untreated controls. A proportion of ATV-treated embryos did not develop ICH and therefore these animals were used as controls for the treatment (ICH-) alongside untreated (UNT) siblings. For both bbh and ATV models, embryos with evident haemorrhages (ICH+) were separated from non-haemorrhaged (ICH-) siblings at ~52 hpf for downstream analyses.

### Locomotion assay

Locomotion was measured at 120 hpf to determine if ICH resulted in a physical phenotype. To remove locomotor function bias, larvae were briefly anesthetised at 72 hpf using 0.02% MS222 in embryo water and selected at random for plating. Following recovery, n=24 larvae were individually transferred to each well of a 24-well plate in 1 ml of fresh methylene-blue-free E3 medium. Cumulative time spent mobile was measured using the DanioVision camera chamber and Ethovision XT software (Noldus, version 11) at room temperature. Analyses were performed on larvae at 72, 96 and 120 hpf. Swimming movement of each individual larva was tracked in the x and y plane for 10 minutes using a white light stimulus to initiate a startle response every 60 seconds. Cumulative time spent swimming was measured from three independent replicates.

### Light sheet microscopy

Transgenic ICH+ and ICH- larvae were imaged using light sheet microscopy to analyse cell death ( *ubiq:*secAnnexinV-mVenus), neutrophils ( *mpo:*GFP) and macrophages ( *mpeg1:*mCherry). At ~72 hpf, randomly selected larvae were anaesthetised using 0.02% MS222 and mounted in 1.5% low-melt agarose (Promega), maintained at room temperature. Images were acquired using a W Plan-Apochromat 20X magnification/1.0 UV-VIS objective for light-sheet microscope (Carl Zeiss Lightsheet Z.1) and processed with ZEN imaging software (version 2.3). Maximum intensity projection (MIP) composites were made from z-stack images and brain regions (excluding the eyes) were analysed for average intensity fluorescence of cells with image background removal using an ImageJ (version 1.52a) macro ( Supplementary File 1). Numbers of fluorescent cells in the brain were also verified by blind manual counts from MIPs. Data were collected from n=6-12 randomly selected larvae per group from 3 independent replicates for ATV studies and verified in two replicates for bbh. For time-lapse recording, MIP composites were stitched from a series of successive z-stack images over a period of 18 hours.

### Statistical analysis

Experimental sample sizes were determined by using power calculations from preliminary data using α=0.05 and β=0.80. All statistical analysis was first performed using GraphPad Prism 7.0 and then verified using R ( R Core Team, 2018) for non-parametric data and subsequent significance values plotted. All data is presented as mean +/- s.d. Linear mixed modelling (LMM) was used to evaluate the effects of independent factors on the continuous dependent variables ( Bates *et al.*, 2015). All factors and interactions were modelled as fixed effects. As there is a lack of independence in fish from the same clutch, “Clutch” was treated as a random effect, modelled with random intercepts for all models. The significance of inclusion of an independent variable or interaction terms were evaluated using log-likelihood ratio. Holm-Sidak post-hocs were then performed for pair-wise comparisons using the least square means ( Lenth, 2016). Homoscedasticity and normality of the Pearson residuals were evaluated graphically using predicted vs residual and Q-Q plots, respectively, and transformations were applied when necessary.

For discrete data and data with non-normal distributions, generalized linear mixed modelling was used (GLMM) ( Bates *et al.*, 2015; Fournier *et al.*, 2012; Skaug *et al.*, 2013). Again “Clutch” was treated as a random effect modelled with random intercepts for all models. Appropriate families were selected based on the data distribution. Numerous families and link functions were evaluated where necessary and the optimal parameters were selected based on the Akaike information criterion (AIC). For mobile or non-mobile (yes/no) data, a logistic regression with cloglog link was selected; for count data (number of dead cells) a negative binomial family was selected. The significance of inclusion of an independent variable or interaction terms were evaluated using log-likelihood ratio. Holm-Sidak post-hocs were then performed for pair-wise comparisons using the least square means ( Lenth, 2016). Pearson residuals were evaluated graphically using predicted vs level plots. All analyses were performed using R ( R Core Team, 2018) ( Supplementary File 3).

## Results

### ATV and bbh mutant models show comparable cerebral bleeding phenotypes

**Figure 1.**
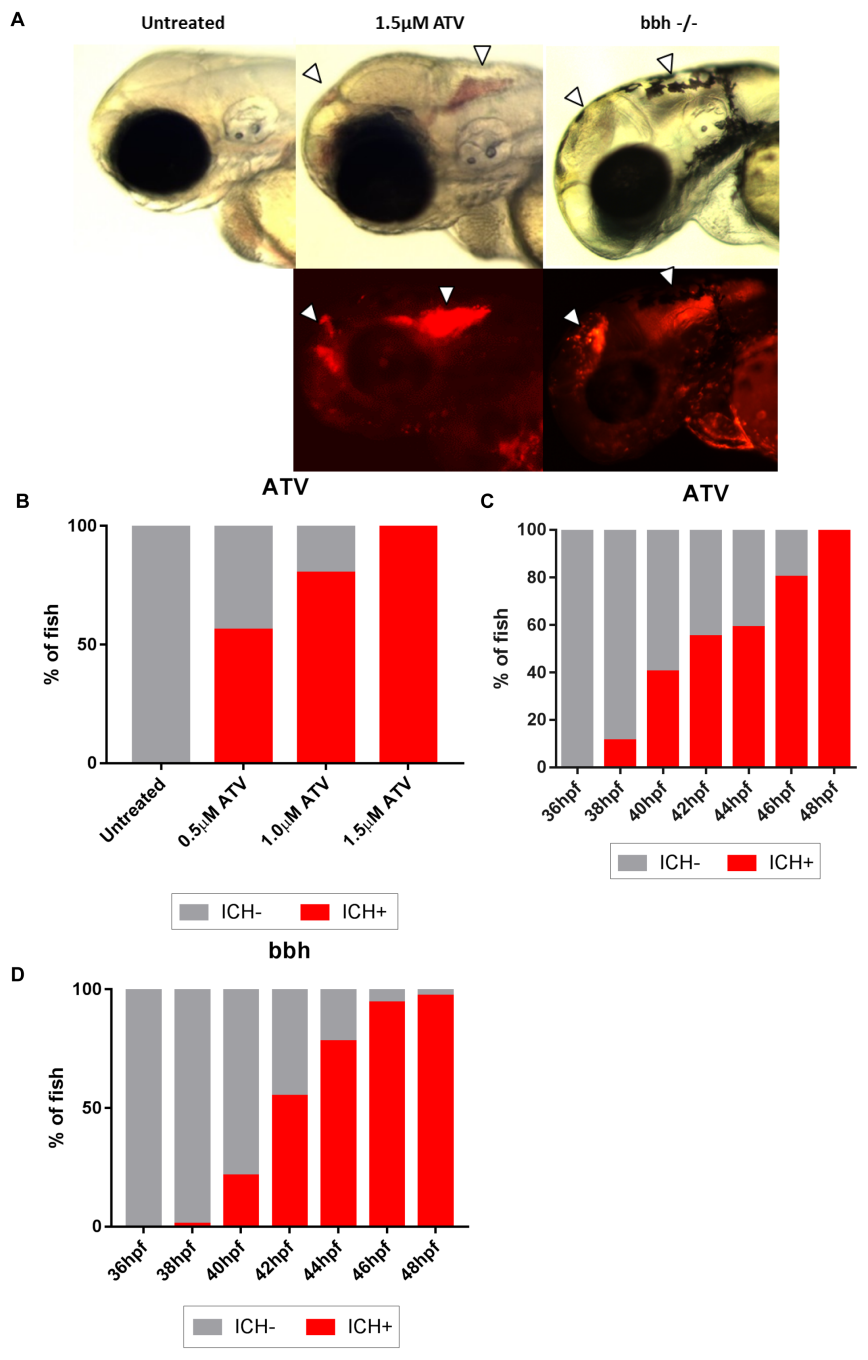
Atorvastatin (ATV)-induced and bubblehead (bbh) mutant intracerebral haemorrhage (ICH) show comparable models of brain-specific bleeding. ( **A**) Brain-specific bleeds were observed in both ATV and bbh models maintained on the transgenic *gata1*:DsRed reporter background using both brightfield (top panels) and fluorescence (bottom panels) microscopy. Bleeds formed in both forebrain and mid-hindbrain regions, as described by others ( Eisa-Beygi *et al.*, 2013) (arrows denotes haemorrhages). Bleeds in bbh mutants are frequently associated with severe cranial oedema making blood pooling more disperse. Original magnification, x20. ( **B**) ATV treatment causes ICH to occur in a dose-dependent manner. ( **C**) Timeline of ICH development in ATV-treated and untreated embryos and ( **D**) bbh homozygotes.

It has been shown that both the ATV and bbh models share similar underlying mechanisms that are responsible for neuroendothelial weakness in the developing larvae and spontaneous cranial vessel rupture ( Eisa-Beygi *et al.*, 2013; Liu *et al.*, 2012). In this study we compared these models in the context of ICH and characterised the pathological outcomes of haemorrhage to develop a new platform for pre-clinical interrogation of post-bleed consequences. Using the translucent nature of the zebrafish embryo we observed brain-specific bleeding non-invasively, using light and fluorescent microscopy ( Figure 1A). Bleeds were observed in fore, mid and hindbrain regions at comparable frequencies, as described by others ( Eisa-Beygi *et al.*, 2013; Liu *et al.*, 2007). ATV absorption induced haemorrhages in a dose-dependent manner when embryos were treated at 24 hpf ( Figure 1B). Homozygous mutant bbh embryos and ATV-treated embryos both exhibited ICH between 38 and 48 hpf ( Figure 1C, D). In homozygous mutant bbh embryos, ICH was frequently accompanied with severe cranial oedema ( Liu *et al.*, 2007) ( Supplementary Figure 3). Wild-type and heterozygous bbh siblings had no haemorrhages and were utilised as ICH-controls.

### ICH in zebrafish larvae results in a quantifiable brain injury

**Figure 2.**
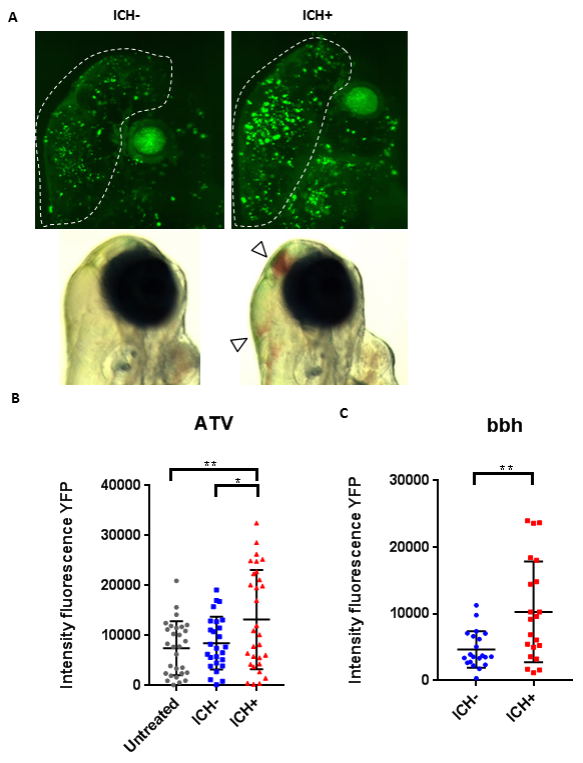
Intracerebral haemorrhage (ICH) in zebrafish larvae results in a quantifiable brain injury. ( **A**) Representative images of the brain injury phenotype in ICH+ larvae (right panels), in comparison to ICH- siblings (left panels), at 72 hpf. Bright-field images (bottom panels) demonstrate the presence of brain bleeds (arrows) in ICH+ larvae. Fluorescent microscopy was performed to visualise cell death in the *ubiq:*secAnnexinV-mVenus reporter line (top panels). Clusters of dying cells were observed in peri-haematomal regions. Images were cropped to brain only regions and analysed for total green fluorescence intensity in round particles bigger than 30 pixels in diameter (white line). ( **B**) Quantification of fluorescent signal in the brains of untreated, ICH- and ICH+ larvae obtained through the ATV model (n=12 per group; 3 independent replicates) at 72 hpf. Significant differences were observed when comparing ICH+ with untreated (**p=0.004) and with ICH- (*p=0.03) siblings. ( **C**) Quantification of fluorescent signal as a read out for annexinV binding in the brains of ICH- and ICH+ larvae obtained through the bubblehead (bbh) model (n=12 per group; 2 independent replicates) at 72 hpf. A significant difference in mVenus fluorescence was observed between ICH+ and ICH- age-matched siblings (**p=0.002). Original magnification, x20.

To determine the pathological consequences of ICH in zebrafish larvae, we next assessed brain injury using a transgenic *ubiq:*secAnnexinV-mVenus cell death reporter line ( Morsch *et al.*, 2015). AnnexinV binding was assessed between 48 and 120 hpf, to characterise the timeline of cell death following an ICH event ( Supplementary Figure 4). We observed peri-haematomal brain-damaged lesions as ‘clusters’ of dying cells formed by 72 hpf, which had receded before 96 hpf ( Supplementary Figure 4). We quantified brain lesions from images taken of bbh siblings at 72 hpf ( Figure 2A). Representative images of ATV model in are given in Supplementary Figure 2. In both ATV and bbh models, bleeding was associated with a significant, two-fold increase in intensity of fluorescent signal in the brain compared to ICH- controls ( Figure 2B, C). These data were verified using blinded, manual counts of annexinV-positive cells from MIP images ( Supplementary Figure 5). Taken together, these data provide convincing evidence that ICH in zebrafish larvae induces a reproducible cell death phenotype that can act as a quantifiable readout of brain injury.

### ICH-induced brain injury results in a quantifiable locomotor deficit in zebrafish larvae

**Figure 3.**
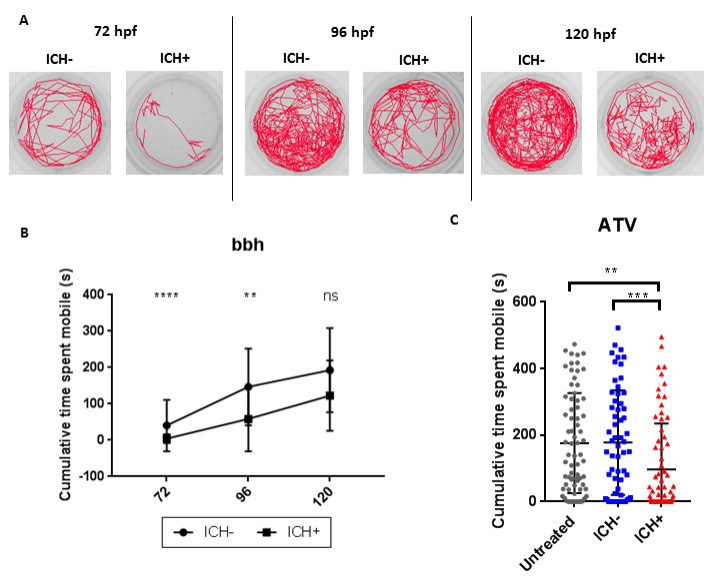
ICH-induced brain injury results in a quantifiable locomotor deficit in bubblehead (bbh) zebrafish larvae. ( **A**) Representative examples of the swimming tracks in ICH- and ICH+ larvae at 72, 96 and 120 hpf. ( **B**) ICH+ larvae exhibited a significant decrease in the cumulative time spent mobile during the 10 minute recording period at both 72 and 96 hpf. Significance was lost at the 120 hpf time point potentially alluding to recovery from brain injury (n=24 larvae per group; 3 independent replicates; ****p=0.00006; **p=0.003 ns p=0.08) ( **C**) Quantification of cumulative time spent moving in untreated and ATV-treated ICH- and ICH+ larvae at 120 hpf. ICH+ larvae exhibited a significant decrease in the cumulative time spent mobile during the 10 minute recording period. Three technical replicates (n=24 larvae per group) were used to calculate s.d from the mean (***p=0.00004, **p=0.0003).

To investigate whether ICH-induced brain injury was associated with a locomotor deficit, as frequently seen in stroke patients ( Kloter *et al.*, 2011; Saulle & Schambra, 2016), we tracked swimming behaviour between 72 and 120 hpf. Larvae were analysed across 3 days to account for the improvement in baseline swimming performance associated with increasing age. Representative tracks are shown in Figure 3A. ICH+ larvae spent significantly less time swimming in the cumulative time spent mobile during the 10 minute recording period at both 72 and 96 hpf compared to ICH- siblings, implying a persistent physical deficit ( Figure 3B). Although a reduction was observed in ICH+ larvae at 120 hpf, this was not significantly different to ICH- siblings (p=0.08). However ATV-treated larvae assayed at 120 hpf did show a significant reduction in swimming in ICH+ larvae and not in controls. Comparable motility phenotypes in both models imply that the swimming deficit is due to cerebral bleed and not statin treatment. Reproducing these results in both models suggests that the impairment in locomotion is not caused by any one mechanistic factor but due to ICH itself.

### ICH-induced brain injury in zebrafish larvae initiates an innate immune response

**Figure 4.**
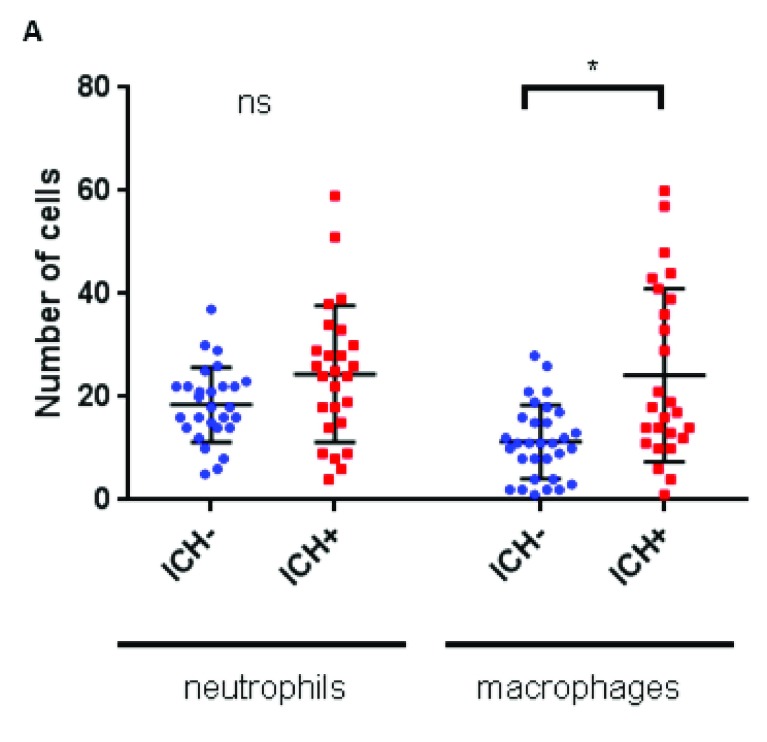
Intracerebral haemorrhage (ICH) initiates an innate cellular immune response in the zebrafish larval brain. Numbers of leukocytes quantified within the brains of *mpo:*GFP; *mpeg1:*dsRed double transgenic larvae (n=8 per group; 2 independent replicates) at 72 hpf reveals a significant increase in macrophages (*p=0.01), but not neutrophils (p=0.5), in response to ICH.

In order to determine whether ICH in zebrafish larvae initiated inflammation at the cellular level, neutrophils and macrophages in the brain were quantified using the *mpo*:GFP and *mpeg1*:mCherry reporter lines. At 72 hpf, the number of macrophages increased significantly, doubling in the brains of ICH+ larvae compared to ICH- siblings ( Figure 4). At the same time point, neutrophil numbers were greater in ICH+ larvae than ICH- larvae; however, the difference did not reach significance.

### Activated macrophage cells show a phagocytic response to the brain lesion

**Figure 5.**
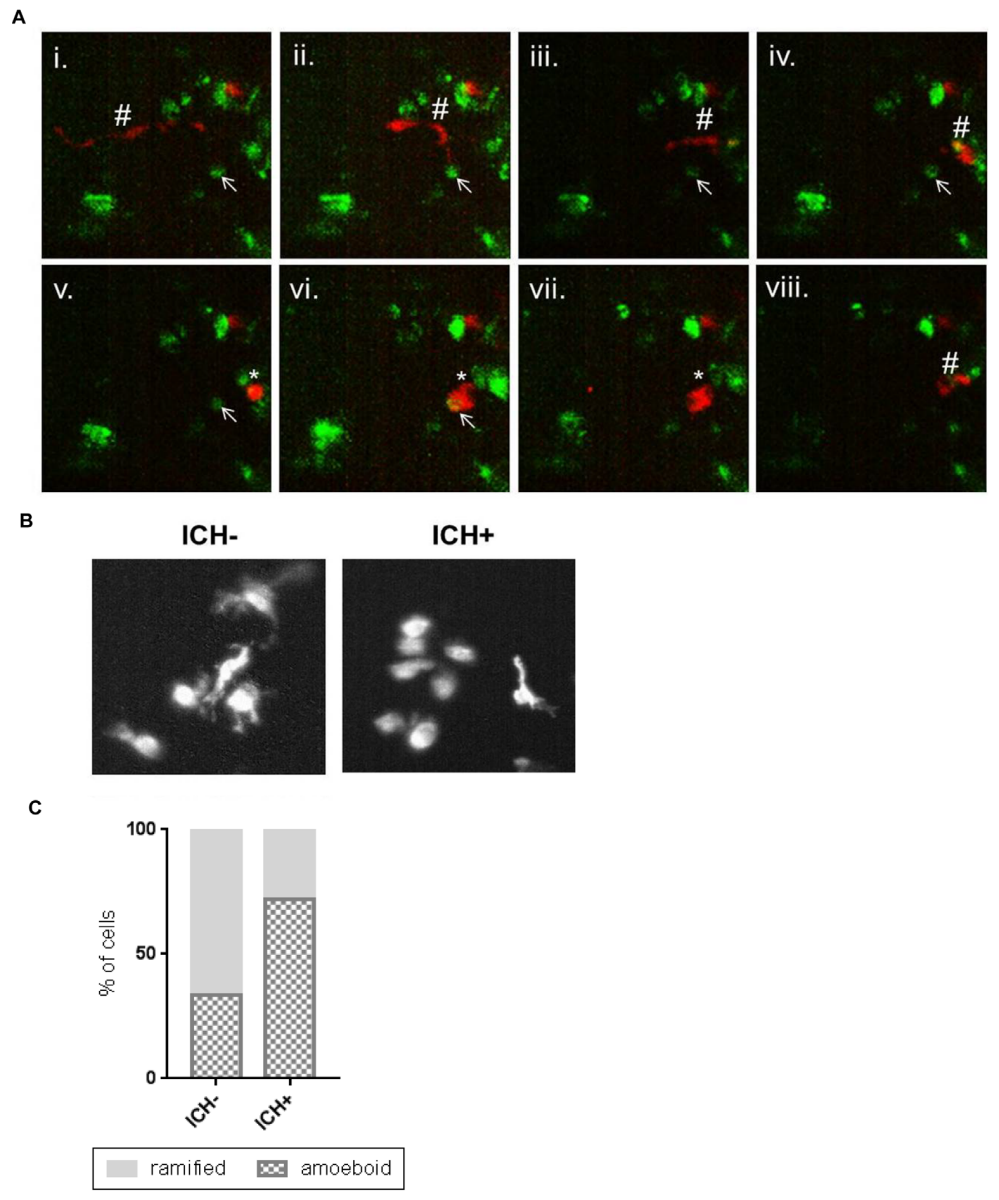
Activated macrophage cells show a phagocytic response to the brain lesion. ( **A**) Representative time-lapse stills (from Supplementary Video 1) showing a ramified patrolling macrophage migrating towards an annexinV positive cell (i - vi). The macrophage acquired an amoeboid morphology (v) before phagocytosing the annexinV-positive cell (vi, vii). After phagocytosis the macrophage resumes a ramified morphology and migrates away and the annexinV-positive cell can no longer be seen (viii). Ramified macrophage (#), annexinV positive cell (arrow), amoeboid macrophage (*). ( **B**) Representative images of *mpeg1*-positive cells in the intracerebral haemorrhage (ICH)- and ICH+ larval brain exhibiting amoeboid and ramified morphologies. ( **C**) An increased proportion of amoeboid (phagocytic) and decreased proportion of ramified (inactive) macrophages was observed in ICH+ brains in comparison to ICH- siblings.

We investigated the phagocytic response of activated macrophages to the brain lesion sites in *ubiq:*secAnnexinV-mVenus; *mpeg1*:mCherry ICH+ larvae using real time microscopy. The formation of a new brain lesion site was recorded between 55 and 65 hpf ( Supplementary Video 1) and macrophages were seen migrating to sites of injury and actively phagocytosing annexinV positive dying cells ( Figure 5A). We next analysed total macrophage activity within the brain, using morphology as an indication of phagocytic activation. Amoeboid, rounded cells were considered phagocytic and ramified cells considered inactive, as previously defined ( Morsch *et al*., 2015). We found a substantial increase in the proportion of activated, amoeboid macrophages in the ICH+ brain compared to ICH- siblings ( Figure 5B, C). These results indicate that the innate immune response to ICH can be examined at the cellular level in zebrafish ICH models using real-time microscopy.

All raw data from the present study Data include all raw microscopy images of cell death in Annexin and bubblehead groups; fluorescence intensities, cell counts and motility times for ATV and bubblehead groups; and leukocyte cell counts. Click here for additional data file. Copyright: © 2018 Crilly S et al. 2018 Data associated with the article are available under the terms of the Creative Commons Zero "No rights reserved" data waiver (CC0 1.0 Public domain dedication).

## Discussion

Here, we reveal that ICH in zebrafish larvae induces quantifiable pathological and inflammatory consequences that mimic aspects of human pathophysiology. Several groups have previously described the mechanisms underlying neurovascular instability in zebrafish larval models of ICH ( Buchner *et al.*, 2007; Eisa-Beygi *et al.*, 2013; Liu *et al.*, 2007), and drug intervention studies have attempted to identify compounds that can inhibit cerebral bleeding ( Li *et al.*, 2017; Yang *et al.*, 2017). However, the relevance and translational impact of intervention before onset of haemorrhage is unclear. As such, we focussed our attention on characterising the pathological and immunological consequences of blood in the brain in zebrafish models, which we consider to be a more realistic therapeutic target.

**Figure 6.**
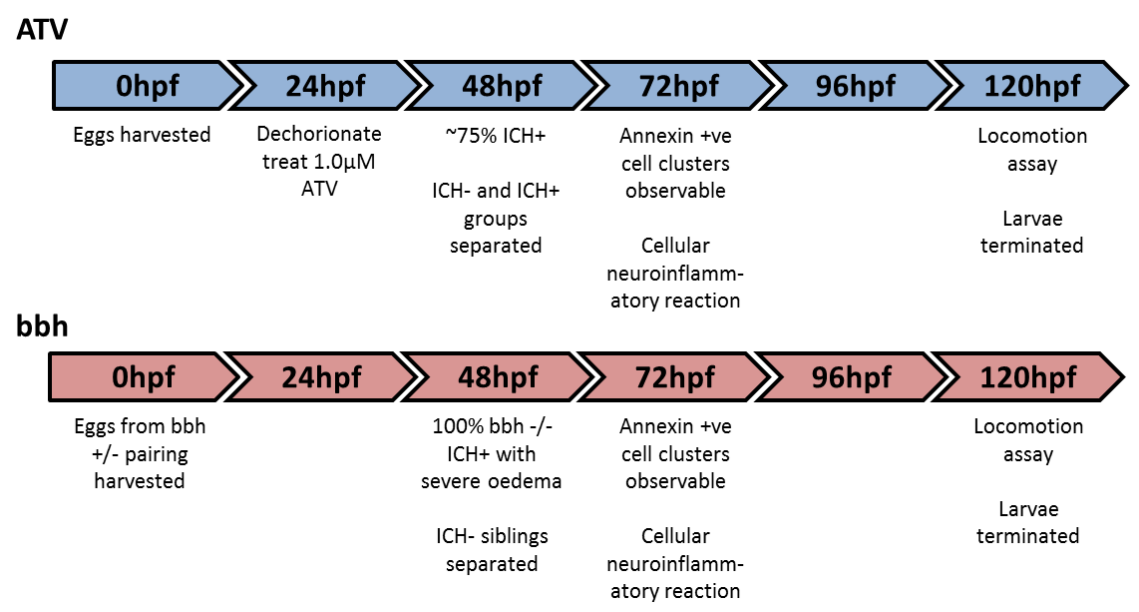
Graphic of experimental timeline to characterise brain injury, locomotor and neuroinflammatory outcomes. ICH, intracerebral haemorrhage; bbh, bubblehead.

In general, ICH can be predominantly considered as a disorder associated with older age. Consequently, it is remarkable that the phenotypes observed in developing zebrafish recapitulate those associated with the aged human brain, indicating the innate injury response to blood in the brain is both evolutionarily conserved between species and analogous during development and adulthood. Zebrafish models have been employed in other neurological and neuropsychiatric disease investigation, including epilepsy, schizophrenia, Alzheimer’s and Parkinson’s disease, because of these conserved fundamental mechanisms and behaviours ( Fontana *et al.*, 2018; Vaz *et al.*, 2018). Although these models present their own limitations, the use of non-invasive *in vivo* imaging, ease of genetic manipulation and availability of large sample sizes offers new insights and overcomes some of the common restrictions associated with rodent models. Given that ICH occurs spontaneously using non-invasive techniques in zebrafish, it can be argued this system more accurately models some aspects of the human condition than the most commonly used rodent models ( Andaluz *et al.*, 2002; Rosenberg *et al.*, 1990). The use of rodents has not been successful in terms of identifying translatable therapeutic targets for ICH. As such, we propose that the post-ICH pathologies presented in this study ( Figure 6) represent an alternative, complementary platform for pre-clinical ICH research and future drug discovery.

The transferability of employing these zebrafish larval models would address the 3Rs welfare issue of prolonged severe surgical procedures in mammals by replacing some of these animals with zebrafish larvae of unprotected status. We have used equipment and procedures that are commonplace and would be relatively simple to adopt in other labs. We would like to see these models used to determine the translatability of drugs that prevent cerebral bleeding in zebrafish ( Huang *et al.*, 2018; Yang *et al.*, 2017), to investigate the clinical relevance of post-ICH treatment. We also propose these endpoint assays can be used to develop medium/high-throughput drug screening to identify new compounds for pre-clinical investigation.

In humans, an influx of blood into the brain causes primary brain injury through neuronal death and cell necrosis, inducing a secondary phase of injury triggered by the production of inflammatory mediators and innate immune cell migration towards the site of injury ( Mracsko & Veltkamp, 2014). We show that cerebral bleeding in zebrafish larvae causes an increase in cell death in the brain not shown before in zebrafish haemorrhage models. It has been established that following ICH neuronal cells die by different death mechanisms including ferroptosis and necroptosis ( Li *et al.*, 2018; Zille *et al.*, 2017) and so determining total cell death by only using one marker, Annexin V may limit the information attained in this study. Further studies into the mechanisms of cell death involved after ICH, and different approaches to inhibit these processes may reveal treatment strategies that have not yet been elucidated in the rodent model, and will be a focus for future studies. The observed brain lesion was associated with a physical impairment, as observed by a reduction in swimming ability, which we propose is either due to a defect in stimulus perception or through a motor deficiency. This impairment is seen to begin to recover at 3 days post injury, suggesting the zebrafish larval model system as a useful tool for further investigation to reveal recovery processes responding to brain cell death. Importantly, these observations mimic outcomes that are exhibited in rodent models ( MacLellan *et al.*, 2008) and ICH patients ( Saulle & Schambra, 2016). In clinical presentation of ICH the initial haematoma mass effect and increase in intracranial pressure exacerbates brain damage, compressing surrounding structures ( Xi *et al.*, 2006) and increases risk of death following ICH ( Yang *et al.*, 2015). In zebrafish larvae, cerebral oedema is regularly associated with ICH ( Kasher *et al.*, 2015; Liu *et al.*, 2007). However without a fully developed cranium, oedema is unlikely to result in the same injury severity seen in humans, which may be one limitation of this particular model system.

An increase in recruitment and activation of macrophages in the brain was also observed in zebrafish following haemorrhage corresponding to time of cell death. A trend towards increased recruitment of neutrophils was also observed, but this result did not reach statistical significance. It is possible that a rapid temporal neutrophil response was not fully captured during our analytical time frame. However, it has been shown that neutrophils are not involved in the clearance of cellular debris in a zebrafish larval model of brain injury ( van Ham *et al.*, 2014), suggesting neutrophils are less vital to early injury responses in the brain than macrophages. It remains unclear whether activation of macrophages post-ICH is overall beneficial or deleterious in the short term after injury. Pro-inflammatory cells contribute to the breakdown of the blood-brain barrier ( Abbott, 2000); however, phagocytic cells promote clearance of red blood cells and tissue debris, which occurs from 7 days post haemorrhage in rodent models of ICH ( Mracsko & Veltkamp, 2014; Yang *et al.*, 2016b). Early responses to laser-induced cerebral bleeding in zebrafish have shown that macrophages are essential for vessel repair ( Liu *et al.*, 2016) and in zebrafish stab wound brain injury models, inflammation is necessary for regeneration and recovery ( Kyritsis *et al.*, 2012). Studies show that polarisation of macrophages to M1-like and M2-like states change over time in rodent models of ICH in response to brain damage molecules ( Wan *et al.*, 2016; Yang *et al.*, 2016a) and some drugs in clinical trials target microglial polarisation in an attempt to prevent the pro-inflammatory phenotype ( Lan *et al.*, 2017). *In vivo* imaging of ICH-induced inflammatory processes has barely been explored ( Mracsko & Veltkamp, 2014), and so the translucent nature of zebrafish larvae and availability of transgenic lines offer an accessible model for further interrogation. Observations of cellular interactions within whole, intact rodent brains at ongoing time points are currently not possible, therefore utilising the zebrafish system will allow us to learn more about leukocyte behaviour after spontaneous ICH. Furthermore, immune responses observed in real time in this system will have been elicited by spontaneous vessel rupture and not as an artefact of ICH surgery or *ex vivo* analysis ( Kirkman *et al.*, 2011; Xue & Del Bigio, 2003). For better translation of therapies from pre-clinical to patients, understanding of early innate immune responses to spontaneous bleeding needs to be improved, thus zebrafish offer a powerful resource to facilitate this.

In conclusion, given the advantages associated with zebrafish larvae and the potential for a 3Rs approach to pre-clinical stroke research, we propose that this model organism can provide critical insight into ICH pathophysiology during the early phases of injury and offers a future platform for drug discovery.

## Data availability

The data referenced by this article are under copyright with the following copyright statement: Copyright: ï¿½ 2018 Crilly S et al.

Data associated with the article are available under the terms of the Creative Commons Zero "No rights reserved" data waiver (CC0 1.0 Public domain dedication). 

**Dataset 1. All raw data from the present study.** Data include all raw microscopy images of cell death in Annexin and bubblehead groups; fluorescence intensities, cell counts and motility times for ATV and bubblehead groups; and leukocyte cell counts. DOI: https://doi.org/10.5256/f1000research.16473.d220415 ( Crilly *et al*., 2018).

## Supplementary material

**Supplementary File 1. Full detail protocol for experimental methodology.**

**Supplementary File 2. Completed ARRIVE guidelines checklist.**

**Supplementary File 3. Supplementary statistical analysis.**

**Supplementary Figure 1. HMG-CoA inhibition and β-pix mutation induces neurovascular weakness through the same pathway, ultimately preventing actin cytoskeleton remodelling and formation of tight junctions resulting in ‘leaky’ endothelial junctions in the developing neurovasculature.**

**Supplementary Figure 2. Representative images of the brain injury phenotype in ATV treatment model of untreated (UNT) (left panels), ATV-treated control siblings without haemorrhage (ICH-) (centre panels) and haemorrhaged larvae (ICH+) (right panels)**

**Supplementary Figure 3. Severe cranial oedema (affected regions denoted by hashtags) associated with cranial bleeds in ‘bubblehead’ homozygous mutants (right panel) and subsequently exhibit more dispersed bleeds (arrows) compared to heterozygous and wild-type intracerebral haemorrhage (ICH) (left panel).**

Original magnification, x20.

**Supplementary Figure 4. Representative images of the brain injury phenotype in intracerebral haemorrhage (ICH)+ larvae (bottom panels), in comparison to ICH- age-matched siblings (top panels), at 48-120 hpf. **

Fluorescent microscopy was performed to visualise cell death in the ubiq:secAnnexinV-mVenus reporter line to determine the timeline of cell injury following an ICH event. Original magnification, x20.

**Supplementary Figure 5. Manual counts of fluorescent cells from blinded analysis of the ubiq:secAnnexinV-mVenus reporter line in atorvastatin (ATV) and bubblehead (bbh) models show a significant increase in intracerebral haemorrhage (ICH)+ (**p=0.0097, ###p=0.0005).**

**Supplementary Video 1. A time lapse recording of phagocytosis occurring in an intracerebral haemorrhage+ ubiq:secAnnexinV-mVenus;mpeg1:mCherry larvae between 55 and 72 hpf.**

Cluster of dying cells (green annexinV positive) in forebrain visible at 55 hpf and active macrophages (red) can be observed phagocytosing cells. A second cluster in the mid brain develops over recording period and macrophages can be seen migrating to the new lesion site. Original magnification, x20.
