# Supplementary material for: Using zebrafish larval models to study brain injury, locomotor and neuroinflammatory outcomes following intracerebral haemorrhage
Source: F1000Res. 2018 Nov 8;7:1617. Originally published 2018 Oct 8. [Version 2] doi: 10.12688/f1000research.16473.2 (PMC6234746; doi:10.12688/f1000research.16473.2)
Supplement: Supplementary file 2 [file f1000research-7-18516-s0001.tgz › 8016d27e-13ed-4893-b401-0d2cc7527fbf_Sup_file_1.docx]

**Using zebrafish larval models to study brain injury, locomotor and neuroinflammatory outcomes following intracerebral haemorrhage**

Siobhan Crilly, BSc^1^ [0000-0003-1046-9884](https://orcid.org/0000-0003-1046-9884), Alexandra Njegic, MRes^2^, Sarah E. Laurie^1^, Elisavet Fotiou, MRes^2^, Georgina Hudson, MSc^1^, Jack Barrington, BSc^1^, Kirsty Webb, MRes^2^, Helen L. Young, PhD^3^, Andrew P. Badrock, PhD^4^, Adam Hurlstone, PhD^3^, Adrian R. Parry-Jones, PhD^2^, Stuart M. Allan, PhD^1*^, Paul R. Kasher, PhD^1*^ [0000-0002-9213-502X](https://orcid.org/0000-0002-9213-502X)

**^1^**Division of Neuroscience and Experimental Psychology, School of Biological Sciences, **^2^**Division of Cardiovascular Sciences, School of Medical Sciences, **^3^**Division of Cancer Sciences, School of Medical Sciences, **^4^**Division of Evolution and Genomic Sciences, School of Biological Sciences, Faculty of Biology, Medicine and Health, Manchester Academic Health Science Centre, University of Manchester, Michael Smith Building, Oxford Road, Manchester, M13 9PT, UK.

* These authors contributed equally to this article.

Correspondence: Dr Paul Kasher, E-mail: [paul.kasher@manchester.ac.uk](mailto:paul.kasher@manchester.ac.uk)

Telephone: +44 161 306 8059

# Supplementary File 1

# Protocols

## Zebrafish breeding and maintenance

Zebrafish were raised and maintained at The University of Manchester Biological Services Unit under standard conditions as previously described (Westerfield, 2000). Adult zebrafish husbandry was approved by the University of Manchester Animal Welfare and Ethical Review Board and carried out specifically for eggs to be used for experimentation. All experiments were performed in accordance with U.K. Home Office regulations (PPL:70/9091). Fertilized embryos were collected from natural spawning and incubated at 28°C in standard E3 embryo medium and staged according to standard guidelines (Kimmel et al., 1995). At experiment end zebrafish larvae were terminated prior to protected status using overdose of MS-222 anaesthesia and freezing. A visual representation of the experimental timeline is available below.

### Reagents

E3 medium 60x: 5mM NaCl, 0.17mM KCl, 0.33mM CaCl2, 0.33mM MgSO4, 10-15 % Methylene Blue

MS-222: 4g tricaine powder, 500ml of dH_2_O, 10 mls of 1 M Tris (pH 9). Adjust pH to ~7

**0hpf**

**24hpf**

**48hpf**

**72hpf**

**96hpf**

**120hpf**

Eggs harvested

Dechorionate treat 1.0µM ATV

~75% ICH+

ICH- and ICH+ groups separated

Annexin +ve cell clusters observable

Cellular neuroinflamm-atory reaction

Imaged larvae terminated

Locomotion assay

Larvae terminated

**ATV**

**bbh**

**0hpf**

**24hpf**

**48hpf**

**72hpf**

**96hpf**

**120hpf**

100% bbh -/- ICH+ with severe oedema

ICH- siblings separated

Eggs from bbh +/- pairing harvested

Annexin +ve cell clusters observable

Cellular neuroinflamm-atory reaction

Imaged larvae terminated

Locomotion assay

Larvae terminated

## Breeding

Day 0

Set up breeding boxes for adult pairs of zebrafish. Bubblehead heterozygous mutant incross will produce homozygous mutant offspring that will haemorrhage, and heterozygous/wildtype offspring that will not.

Day 1 – 0 hpf

Collect eggs from breeding pair into 30mls E3 medium in a petri dish and incubate at 28°C. Stage the embryos according to standard guidelines (Kimmel et al., 1995).

## Atorvastatin treatment

Day 2 – 24 hpf

Count 100 embryos and transfer them into two clean petri dishes filled with 30ml of E3 medium. Numbers required for experimentation can be adjusted accordingly. 1.0µM ATV treatment will result in ~25% ICH- larvae. Dechorionate the embryos using sharp thin forceps (Liang, 2011). Leave one petri dish untreated and remove 60µl of embryo water from the other and replace with 60µl of 0.5mM atorvastatin. At a 0.5mM stock concentration this results in a final concentration of 1µM. Atorvastatin incubation can be longer than 24h if necessary as ATV causes no further developmental issues.

### Reagents

Atorvastatin (Sigma-Aldrich, PZ0001) was solubilised in distilled water (3mg into 10 mls) to make a 0.5mM stock solution. Incubate overnight at room temperature with agitation as solubilisation takes some time. Do not use DMSO. Solution was aliquoted and stored at -20°C.

E3 medium.

### Equipment

Petri dishes, stripette and pipette boy, p200 pipette, Pasteur pipettes, forceps

## Separating ICH- and ICH+ populations

Day 3 – 48 hpf

Bubblehead larvae from a heterozygous incross and 1.0µM ATV treated larvae will both result in some ICH+ and some ICH- larvae. The groups can be separated manually at 2dpf using a light stereomicroscope and a pipette. We chose to move ICH+ larvae (examples below; red/brown colouration in brain) into clean petri dishes with E3 medium using a Pasteur pipette. ICH- larvae were also moved into fresh medium after the separation process. This can be done under anaesthetic however at this age the larvae do not move much and with practice identifying haemorrhages becomes very quick and easy so anaesthesia can be avoided.


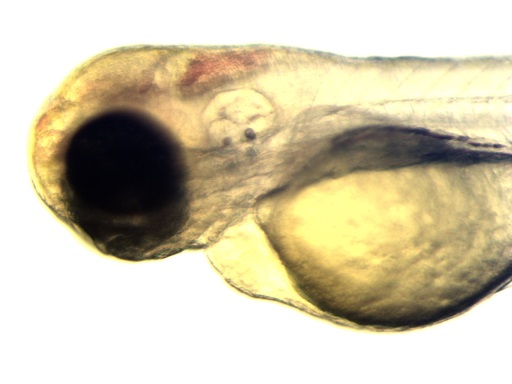

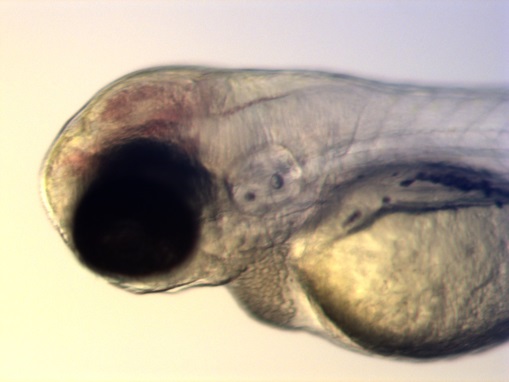

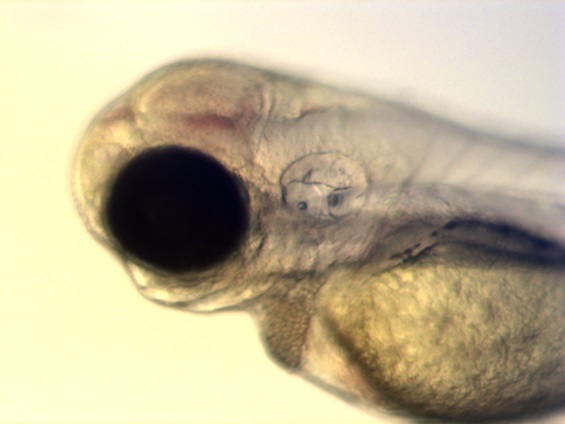


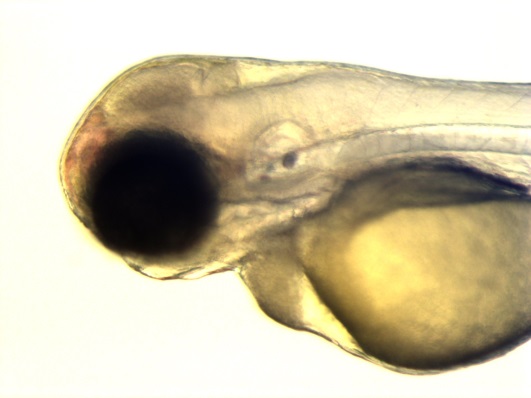

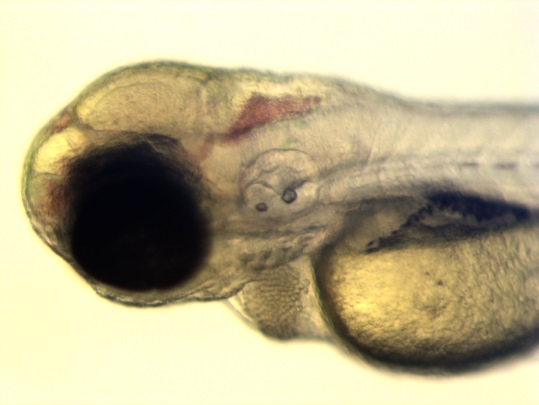

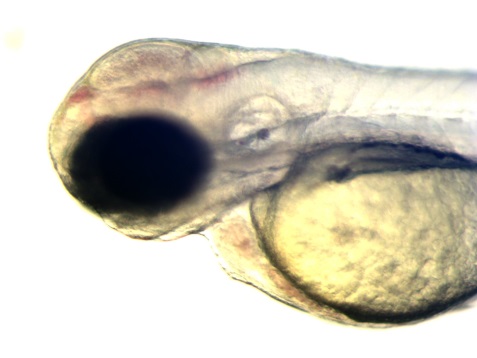


## Cell death and leukocyte analysis

Day 4 – 72 hpf

At 72 hpf larvae were selected at random using a plastic Pasteur pipette (not using a microscope to bias animal selection) from each experimental group and anaesthetised with 0.02% MS222 in E3 embryo water. Larvae were moved to a clean petri dish in a single droplet, excess liquid removed, and 1.5% low-melt agarose added. The larvae were mounted head first into the smallest mounting capillary suitable for and supplied by Zeiss (Lightsheet Z.1). Further mounting information and details are available at the Zeiss website (Zeiss, 2013). Images acquired using a W Plan-Apochromat 20X/1.0 UV-VIS objective for light-sheet microscope (Carl Zeiss Lightsheet Z.1) and processed with ZEN imaging software. Maximum intensity projection (MIP) composites were made from z-stack images and brain regions (excluding the eyes) were analysed for average intensity fluorescence of cells with image background removal using an ImageJ macro (appendix 1). Data was collected from n=12 larvae from each group, from three different experimental repeats and data pooled for analysis.

Upon completion of imaging the larvae were ejected from the sample mount into an overdose of MS-222 and then put into -20°C overnight.

### Reagents

1.5% low melt agarose – 0.15g of low melt agarose (Promega) was dissolved in 10 mls of E3 medium – methylene blue in a microwave and kept at 45°C until use.

MS-222 and E3 medium.

### Equipment

Carl Zeiss Lightsheet Z.1, heat block, petri dishes, pasteur pipettes, capillary mounts

## Locomotion assay

Day 4 – 72 hpf

Larvae were briefly anaesthetised (10 minutes) for random selection for a locomotion assay. Anaesthesia removes any bias that might be included in selection i.e less evasive behaviour and easier capture. Larvae destined for locomotion assay were transferred into clean petri dishes of E3 medium and incubated at 28°C.

Day 6 – 120 hpf

Larvae selected at 72 hpf were transferred into E3 medium –methylene blue, and plated one larvae into 1 ml per well of a 24 well plate using a P1000 pipette tip. Cut end of tip off to minimise damage to larva. Plates were loaded into the DanioVision camera chamber and assayed for 10 minutes at room temperature. Ethovision XT software (Noldus) was used to run a white light startle routine that changed every 60 seconds to increase spontaneous swimming, and recorded the cumulative time spent mobile for each larvae. Data was collected from n=24 larvae from each group, from three different experimental repeats and data pooled for analysis.

Upon assay completion larvae were transferred from the assay plates into an overdose of MS-222 and then put into -20°C.

### Reagents

E3 medium and MS-222

### Equipment

DanioVision camera chamber, 24 well plates, pipettes

## Appendix 1

### ImageJ Macro for annexin fluorescence analysis

outputFolder = getDirectory("image")

Imagetitle = getTitle;

run("Split Channels");

selectWindow(Imagetitle+" (blue)");

close();

selectWindow(Imagetitle+" (red)");

close();

selectWindow(Imagetitle+" (green)");

//run("Threshold...");

setAutoThreshold("Default dark");

setThreshold(0, 120);

run("Measure");

selectWindow("Results");

saveAs("Measurements", "" + outputFolder + Imagetitle

+"Background.xls");

selectWindow(Imagetitle+" (green)");

//run("Threshold...");

setAutoThreshold("Default");

setThreshold(120, 255);

run("Analyze Particles...", "size=30-10000 show=[Overlay Masks] display");

selectWindow ("Results");

saveAs("Measurements", "" + outputFolder + Imagetitle +".xls");

selectWindow(Imagetitle+" (green)");

close();

## References

KIMMEL, C. B., BALLARD, W. W., KIMMEL, S. R., ULLMANN, B. & SCHILLING, T. F. 1995. Stages of embryonic development of the zebrafish. *Developmental dynamics,* 203**,** 253-310.

LIANG, J. O. 2011. *Zebrafish in the Classroom* [Online]. Available: <http://www.zfic.org> [Accessed 21.08.2018].

WESTERFIELD, M. 2000. The zebrafish book: a guide for the laboratory use of zebrafish. [*http://zfin.org/zf_info/zfbook/zfbk.html*](http://zfin.org/zf_info/zfbook/zfbk.html).

ZEISS, C. 2013. ZEISS Lightsheet Z.1 Sample Preparation.
