# Supplementary material for: Using zebrafish larval models to study brain injury, locomotor and neuroinflammatory outcomes following intracerebral haemorrhage
Source: F1000Res. 2018 Nov 8;7:1617. Originally published 2018 Oct 8. [Version 2] doi: 10.12688/f1000research.16473.2 (PMC6234746; doi:10.12688/f1000research.16473.2)

Crilly et al. 2018 Supplementary Analysis

# Packages

lme4; lmerTest; lsmeans; multcompView; ggplot2

Douglas Bates, Martin Maechler, Ben Bolker, Steve Walker (2015). Fitting Linear Mixed-Effects Models Using lme4. Journal of Statistical Software, 67(1), 1-48. doi:10.18637/jss.v067.i01.

Russell V. Lenth (2016). Least-Squares Means: The R Package
lsmeans. Journal of Statistical Software, 69(1), 1-33.
doi:10.18637/jss.v069.i01


Spencer Graves, Hans-Peter Piepho and Luciano Selzer with help
from Sundar Dorai-Raj (2015). multcompView: Visualizations of
Paired Comparisons. R package version 0.1-7.
https://CRAN.R-project.org/package=multcompView

H. Wickham. ggplot2: Elegant Graphics for Data Analysis.
Springer-Verlag New York, 2016.

Fournier DA, Skaug HJ, Ancheta J, Ianelli J, Magnusson A, Maunder
M, Nielsen A, Sibert J (2012). "AD Model Builder: using automatic
differentiation for statistical inference of highly parameterized
complex nonlinear models." _Optim. Methods Softw._, *27*, 233-249.

Skaug H, Fournier D, Bolker B, Magnusson A, Nielsen A
(2016-01-19). _Generalized Linear Mixed Models using 'AD Model
Builder'_. R package version 0.8.3.3.

# ATV

## Cell Death

### Loading data

data<-read.csv("ATVANNEXDEATHrecoded.csv", header=T)
str(data)

'data.frame': 86 obs. of 5 variables:
 $ Clutch : Factor w/ 3 levels "a","b","c": 1 1 1 1 1 1 1 1 1 1 ...
 $ Factor1 : Factor w/ 2 levels "ICH-","ICH+": 1 1 1 1 1 1 1 1 1 1 ...
 $ Factor2 : Factor w/ 2 levels "Treat","UNT": 2 2 2 2 2 2 2 1 1 1 ...
 $ Data : num 15635 10895 12471 11516 11744 ...
 $ Treatment: Factor w/ 3 levels "ICH-","ICH+",..: 3 3 3 3 3 3 3 1 1 1 ...

### Multi level linear modelling with clutch as a random factor

Because there is a lack of independence between individuals from the same clutch, multi-level linear modelling was performed with clutch as a random factor. The two-factor design has a missing factor combination, namely untreated/ICH+. This lack of balance is not handled well by ordinary least square (OLS) methods, again pointing to the need for multi-level linear modelling.

There was a significant effect of Factor1 (intracerebral hemorrhage (ICH)) and no significant effect ATV treatment independent of its ability to induce ICH.

lme1<-lmer(Data~1 + (1|Clutch), data=data, na.action=na.omit)
lme2<-update(lme1, .~. +Factor1)
lme3<-update(lme2, .~. +Factor2)
lme4<-update(lme3, .~. +Factor2*Factor1)

fixed-effect model matrix is rank deficient so dropping 1 column / coefficient

anova(lme1,lme2,lme3,lme4)

refitting model(s) with ML (instead of REML)

Data: data
Models:
lme1: Data ~ 1 + (1 | Clutch)
lme2: Data ~ (1 | Clutch) + Factor1
lme3: Data ~ (1 | Clutch) + Factor1 + Factor2
lme4: Data ~ (1 | Clutch) + Factor1 + Factor2 + Factor1:Factor2
 Df AIC BIC logLik deviance Chisq Chi Df Pr(>Chisq)
lme1 3 1770.1 1777.5 -882.07 1764.1
lme2 4 1761.3 1771.1 -876.64 1753.3 10.8623 1 0.0009814 ***
lme3 5 1762.7 1774.9 -876.33 1752.7 0.6217 1 0.4304303
lme4 5 1762.7 1774.9 -876.33 1752.7 0.0000 0 1.0000000
---
Signif. codes: 0 '***' 0.001 '**' 0.01 '*' 0.05 '.' 0.1 ' ' 1

### Assumption check

There is no evidence for lack of normality of the residuals, no relationship between fitted value and residual spread and, therefore, the residuals are reasonably homoskedasticity. Therefore, there is not sufficient evidence of any violation of the assumptions.

plot(lme4,col=data$Factor1,pch=as.numeric(data$Clutch)+14,cex=0.6)


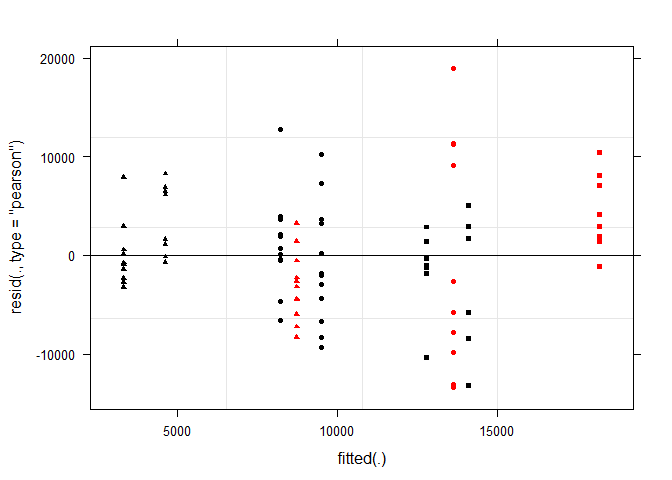


qqnorm(resid(lme4))


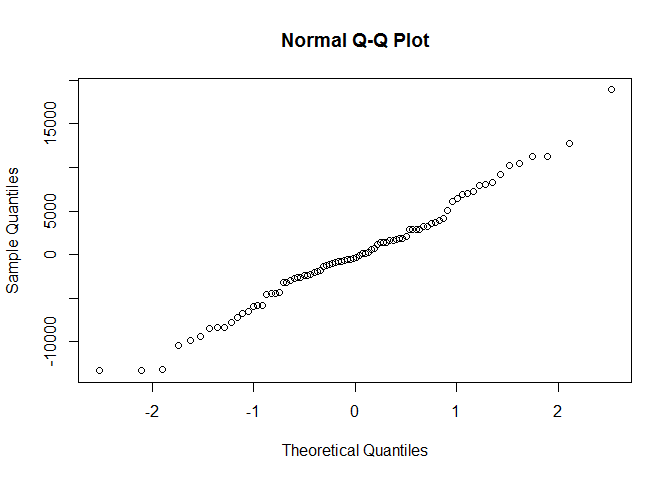


augDat <- data.frame(data,resid=residuals(lme4,type="pearson"),
 fitted=fitted(lme4))
ggplot(augDat,aes(x=Factor1,y=resid,col=Clutch))+geom_point()+geom_boxplot(aes(group=Factor1),alpha = 0.1)+coord_flip()


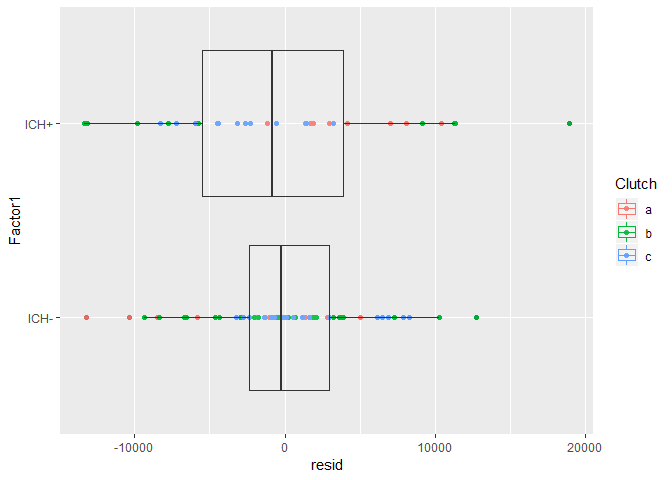


ggplot(augDat,aes(x=Factor2,y=resid,col=Clutch))+geom_point()+geom_boxplot(aes(group=Factor2),alpha = 0.1)+coord_flip()


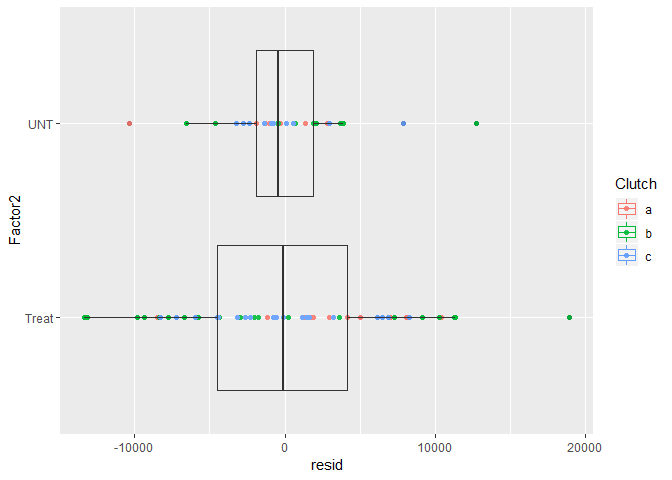


### Post-hoc analysis

Sidák corrected post-hoc analysis was performed using the least-squares estimates. From this we see that the ICH+ group was significantly different to the ICH- Treated and ICH- Untreated groups.

options(scipen=999)
lsmeans <- lsmeans::lsmeans


post <- lsmeans(lme4, pairwise ~ Factor1*Factor2, adjust="none")
posthoc<-summary(post)
comparisons<-c(1,2,4)

p.value<-data.frame(cbind(posthoc$contrasts["contrast"],posthoc$contrasts["p.value"]))

p<-p.value[comparisons,2]
comp<-p.value[comparisons,]
padj<-p.adjust(p, method="holm", n= length(p))
ptable<-data.frame(cbind(comp,padj))
print(ptable, row.names=FALSE)

contrast p.value padj
 ICH-,Treat - ICH+,Treat 0.015920612 0.031841225
 ICH-,Treat - ICH-,UNT 0.438349656 0.438349656
 ICH+,Treat - ICH-,UNT 0.001369473 0.004108418

## Motility

### Loading data

data<-read.csv("ATVmotility.csv", header=T)
str(data)

'data.frame': 204 obs. of 5 variables:
 $ Clutch : Factor w/ 3 levels "a","b","c": 1 1 1 1 1 1 1 1 1 1 ...
 $ Group : Factor w/ 3 levels "ICH-","ICH+",..: 3 3 3 3 3 3 3 3 3 3 ...
 $ Factor1: Factor w/ 2 levels "ICH-","ICH+": 1 1 1 1 1 1 1 1 1 1 ...
 $ Factor2: Factor w/ 2 levels "Treated","UNT": 2 2 2 2 2 2 2 2 2 2 ...
 $ Data : num 1.33 395.6 444.27 24 112.27 ...

## Multi level linear modelling with clutch as a random factor

For the same reasons stated above multi-level modelling was used with clutch as a random factor.

lme1<-lmer(Data~1 + (1|Clutch), data=data, na.action=na.omit)
lme2<-update(lme1, .~. +Factor1)
lme3<-update(lme2, .~. +Factor2)
lme4<-update(lme3, .~. +Factor2*Factor1)
anova(lme1,lme2,lme3,lme4)

Data: data
Models:
lme1: Data ~ 1 + (1 | Clutch)
lme2: Data ~ (1 | Clutch) + Factor1
lme3: Data ~ (1 | Clutch) + Factor1 + Factor2
lme4: Data ~ (1 | Clutch) + Factor1 + Factor2 + Factor1:Factor2
 Df AIC BIC logLik deviance Chisq Chi Df Pr(>Chisq)
lme1 3 2602.7 2612.7 -1298.4 2596.7
lme2 4 2589.4 2602.7 -1290.7 2581.4 15.3128 1 0.0000911 ***
lme3 5 2591.4 2608.0 -1290.7 2581.4 0.0044 1 0.9471
lme4 5 2591.4 2608.0 -1290.7 2581.4 0.0000 0 1.0000
---
Signif. codes: 0 '***' 0.001 '**' 0.01 '*' 0.05 '.' 0.1 ' ' 1

### Assumption check

There is no substantial departure from normality of the residuals, however, there is a relationship between the fitted value and residual spread and, therefore, a lack homoskedasticity. Hence, there is sufficient evidence that the assumptions of the model have been violated, therefore, shrinking transformations were attempted.

plot(lme4,col=data$Factor1,pch=as.numeric(data$Clutch)+14,cex=0.6)


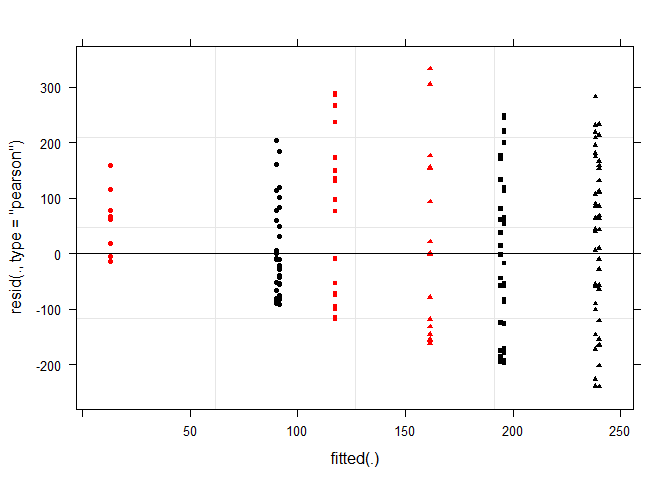


qqnorm(resid(lme4))


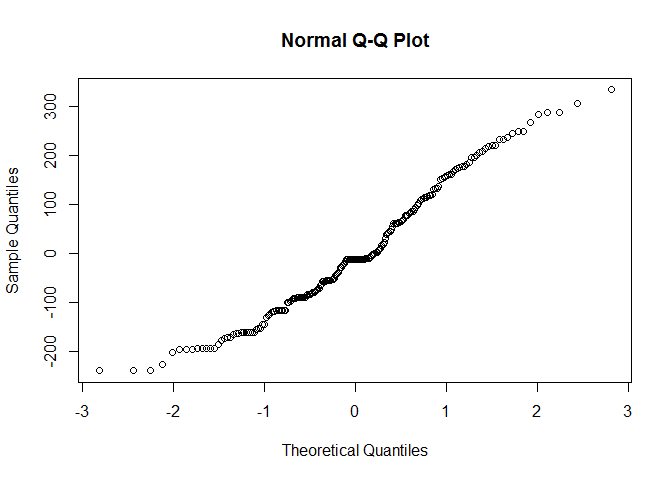


augDat <- data.frame(data,resid=residuals(lme4,type="pearson"),
 fitted=fitted(lme4))
ggplot(augDat,aes(x=Factor1,y=resid,col=Clutch))+geom_point()+geom_boxplot(aes(group=Factor1),alpha = 0.1)+coord_flip()


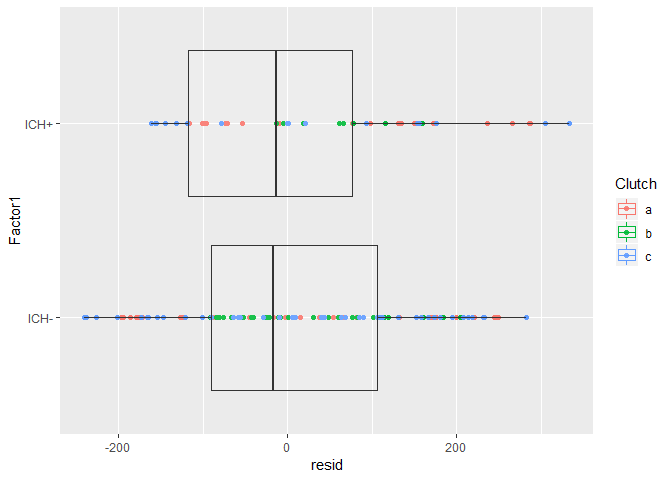


ggplot(augDat,aes(x=Factor2,y=resid,col=Clutch))+geom_point()+geom_boxplot(aes(group=Factor2),alpha = 0.1)+coord_flip()


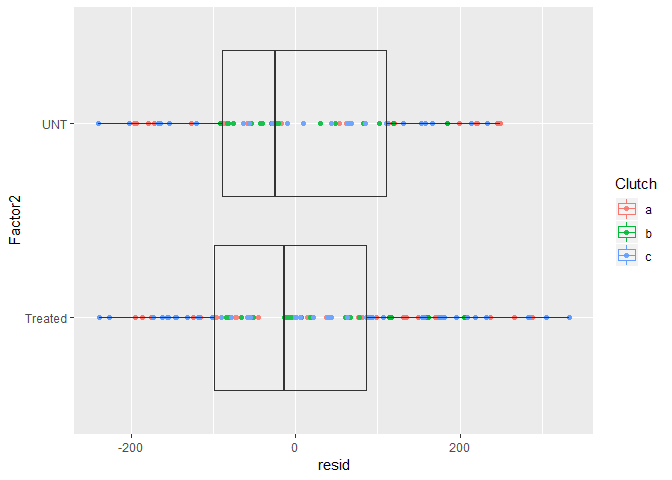


### Transformation of the data prior MLM

Several shrinking transformations were attempted and the square-root transformation was found to be optimal. As above, there was a significant effect of Factor1 (intracerebral hemorrhage (ICH)) and no significant effect ATV treatment independent of its ability to induce ICH.

lme1<-lmer(sqrt(Data)~1 + (1|Clutch), data=data, na.action=na.omit)
lme2<-update(lme1, .~. +Factor1)
lme3<-update(lme2, .~. +Factor2)
lme4<-update(lme3, .~. +Factor2*Factor1)
anova(lme1,lme2,lme3,lme4)

Data: data
Models:
lme1: sqrt(Data) ~ 1 + (1 | Clutch)
lme2: sqrt(Data) ~ (1 | Clutch) + Factor1
lme3: sqrt(Data) ~ (1 | Clutch) + Factor1 + Factor2
lme4: sqrt(Data) ~ (1 | Clutch) + Factor1 + Factor2 + Factor1:Factor2
 Df AIC BIC logLik deviance Chisq Chi Df Pr(>Chisq)
lme1 3 1375.5 1385.4 -684.74 1369.5
lme2 4 1355.2 1368.5 -673.60 1347.2 22.2836 1 0.000002352 ***
lme3 5 1357.0 1373.6 -673.49 1347.0 0.2065 1 0.6495
lme4 5 1357.0 1373.6 -673.49 1347.0 0.0000 0 1.0000
---
Signif. codes: 0 '***' 0.001 '**' 0.01 '*' 0.05 '.' 0.1 ' ' 1

### Assumption check

After the square-root transformation the residuals appear acceptably normally distributed. There is also little evidence of a relationship between fitted value and residual spread and the residuals are reasonably homoskedastic. Therefore, there is not sufficient evidence that any assumption of the model has been violated after the data was transformed.

qqnorm(resid(lme4))


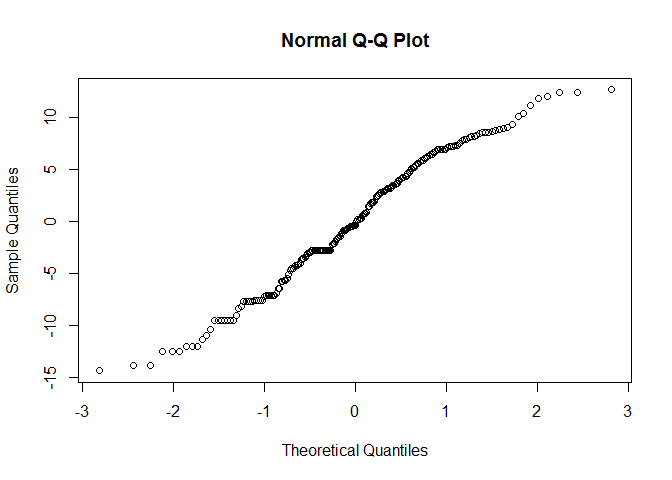


plot(lme4,col=data$Factor1,pch=as.numeric(data$Clutch)+14,cex=0.6)


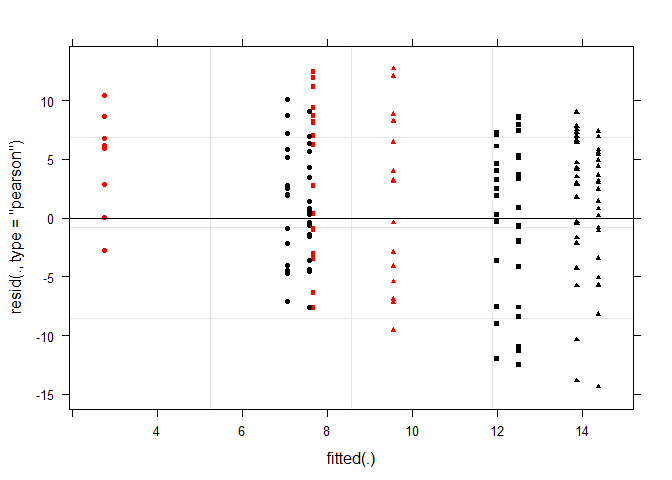


augDat <- data.frame(data,resid=residuals(lme4,type="pearson"),
 fitted=fitted(lme4))
ggplot(augDat,aes(x=Factor1,y=resid,col=Clutch))+geom_point()+geom_boxplot(aes(group=Factor1),alpha = 0.1)+coord_flip()


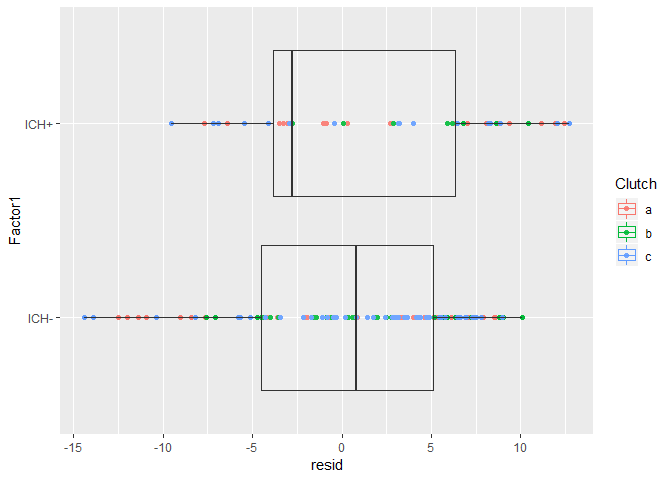


ggplot(augDat,aes(x=Factor2,y=resid,col=Clutch))+geom_point()+geom_boxplot(aes(group=Factor2),alpha = 0.1)+coord_flip()


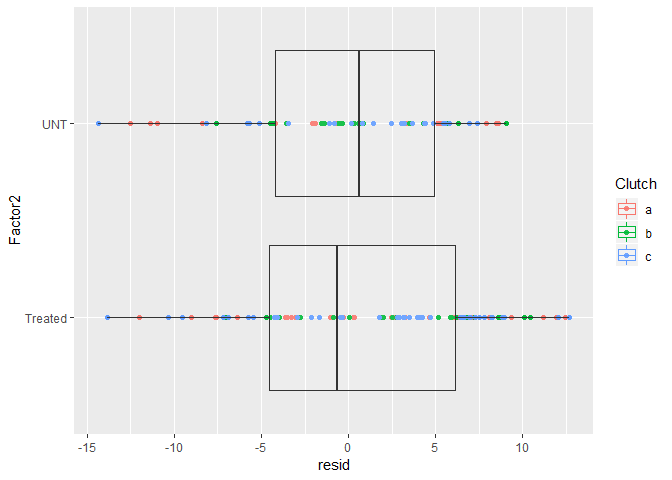


### Post-hoc analysis

Sidák corrected post-hoc analysis was performed using the least-squares estimates. From this the ICH+ group was found to be significantly different to the ICH- Treated and ICH- Untreated groups.

options(scipen=999)
lsmeans <- lsmeans::lsmeans


post <- lsmeans(lme4, pairwise ~ Factor1*Factor2, adjust="none")
posthoc<-summary(post)
comparisons<-c(1,2,4)

p.value<-data.frame(cbind(posthoc$contrasts["contrast"],posthoc$contrasts["p.value"]))

p<-p.value[comparisons,2]
comp<-p.value[comparisons,]
padj<-p.adjust(p, method="holm", n= length(p))
ptable<-data.frame(cbind(comp,padj))
print(ptable, row.names=FALSE)

contrast p.value padj
 ICH-,Treated - ICH+,Treated 0.00018172766 0.00036345533
 ICH-,Treated - ICH-,UNT 0.64960896415 0.64960896415
 ICH+,Treated - ICH-,UNT 0.00001347273 0.00004041819

# BBH

## Annexin cell death

### Loading data

data<-read.csv("bbhannexindeath.csv", header=T)
str(data)

'data.frame': 42 obs. of 3 variables:
 $ Clutch : Factor w/ 2 levels "a","b": 1 1 1 1 1 1 1 1 1 1 ...
 $ Factor1: Factor w/ 2 levels "ICH-","ICH+": 1 1 1 1 1 1 1 1 1 1 ...
 $ Data : num 3516 7064 7380 3393 2787 ...

## Multi level linear modelling with clutch as a random factor

For the same reasons stated above multi-level modelling was used with clutch as a random factor.

lme1<-lmer(Data~1 + (1|Clutch), data=data, na.action=na.omit)
lme2<-update(lme1, .~. +Factor1)

anova(lme1,lme2)

Data: data
Models:
lme1: Data ~ 1 + (1 | Clutch)
lme2: Data ~ (1 | Clutch) + Factor1
 Df AIC BIC logLik deviance Chisq Chi Df Pr(>Chisq)
lme1 3 858.94 864.16 -426.47 852.94
lme2 4 851.26 858.21 -421.63 843.26 9.6818 1 0.001861 **
---
Signif. codes: 0 '***' 0.001 '**' 0.01 '*' 0.05 '.' 0.1 ' ' 1

### Assumption check

There is substantial departure from normality of the residuals, as well as, a relationship between the fitted value and residual spread and, therefore, a lack homoskedasticity. Hence, there is sufficient evidence that the assumptions of the model have been violated, therefore, shrinking transformations were attempted.

plot(lme2,col=data$Factor1,pch=as.numeric(data$Clutch)+14,cex=0.6)


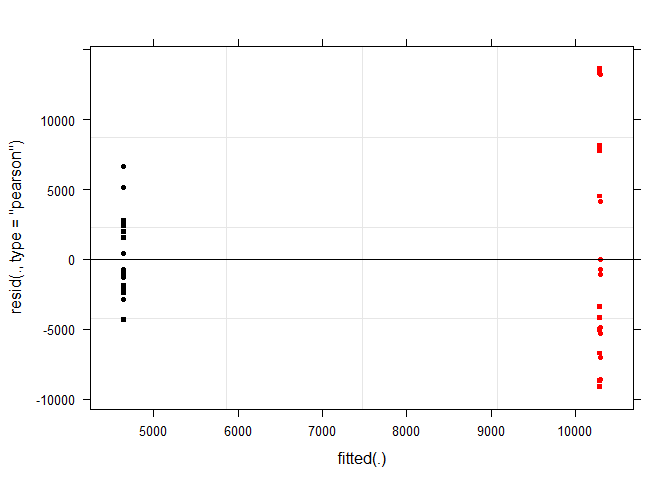


qqnorm(resid(lme2))


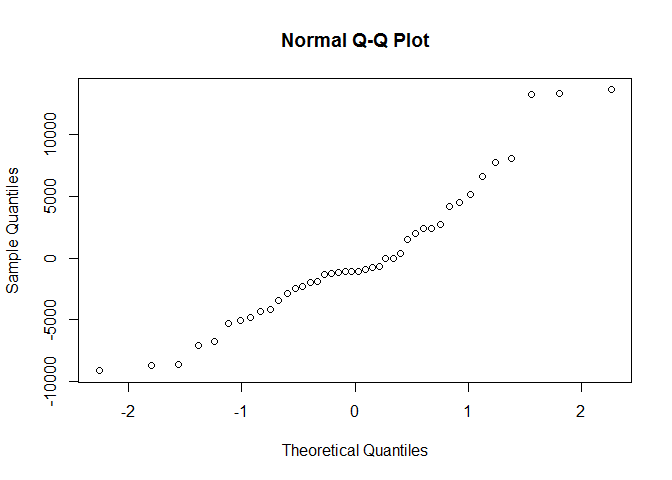


augDat <- data.frame(data,resid=residuals(lme2,type="pearson"),
 fitted=fitted(lme2))
ggplot(augDat,aes(x=Factor1,y=resid,col=Clutch))+geom_point()+geom_boxplot(aes(group=Factor1),alpha = 0.1)+coord_flip()


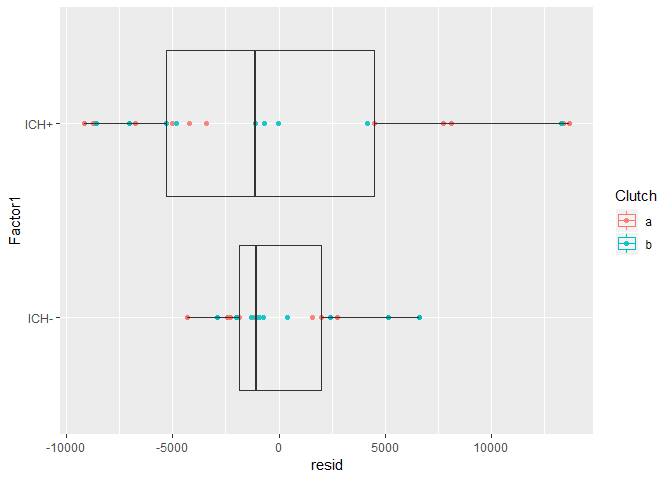


## Following transformation multi level linear modelling with clutch as a random factor

There was a significant effect of ICH on cell death.

lme1<-lmer(sqrt(Data+1)~1 + (1|Clutch), data=data, na.action=na.omit)
lme2<-update(lme1, .~. +Factor1)

anova(lme1,lme2)

Data: data
Models:
lme1: sqrt(Data + 1) ~ 1 + (1 | Clutch)
lme2: sqrt(Data + 1) ~ (1 | Clutch) + Factor1
 Df AIC BIC logLik deviance Chisq Chi Df Pr(>Chisq)
lme1 3 419.69 424.91 -206.85 413.69
lme2 4 412.81 419.76 -202.41 404.81 8.8789 1 0.002885 **
---
Signif. codes: 0 '***' 0.001 '**' 0.01 '*' 0.05 '.' 0.1 ' ' 1

### Assumption check

After the square-root transformation the residuals appear acceptably normally distributed. There is also little evidence of a relationship between fitted value and residual spread and the residuals are reasonably homoskedastic. Therefore, there is not sufficient evidence that any assumption of the model has been violated after the data was transformed.

plot(lme2,col=data$Factor1,pch=as.numeric(data$Clutch)+14,cex=0.6)


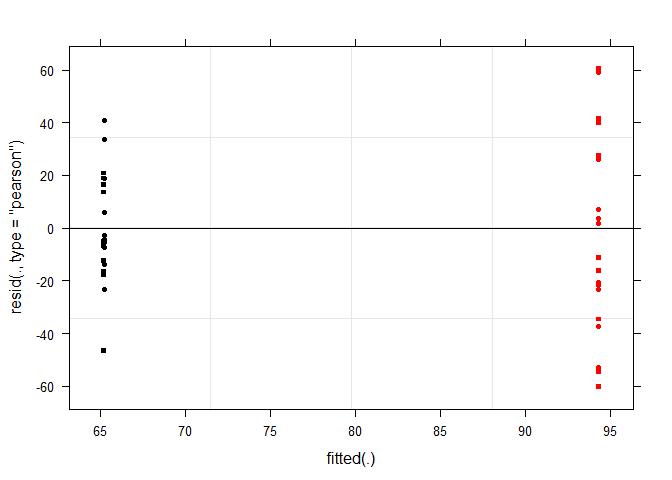


qqnorm(resid(lme2))


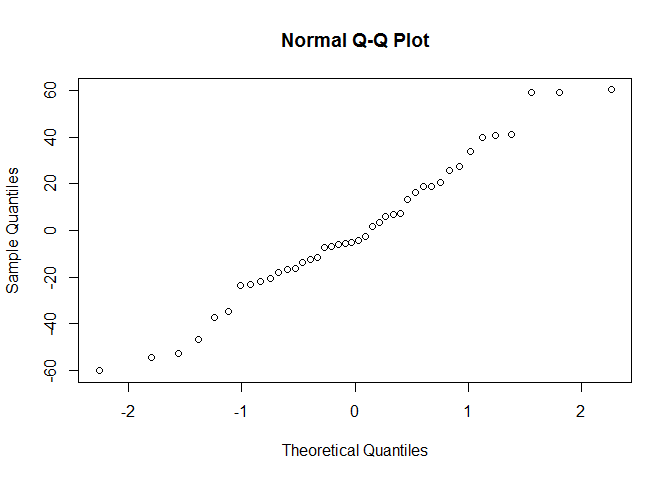


augDat <- data.frame(data,resid=residuals(lme2,type="pearson"),
 fitted=fitted(lme2))
ggplot(augDat,aes(x=Factor1,y=resid,col=Clutch))+geom_point()+geom_boxplot(aes(group=Factor1),alpha = 0.1)+coord_flip()


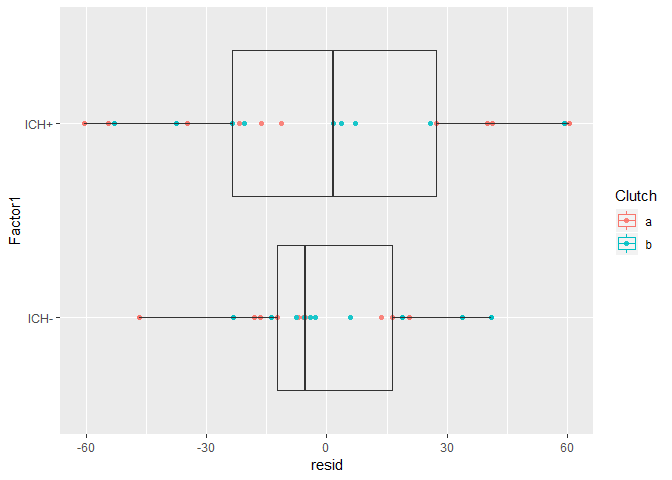


## BBH Motility

### Loading data

data<-read.csv("bbhmotility.csv", header=T)
str(data)

'data.frame': 428 obs. of 5 variables:
 $ Clutch : Factor w/ 3 levels "a","b","c": 1 1 1 1 1 1 1 1 1 1 ...
 $ Factor1 : Factor w/ 2 levels "ICH-","ICH+": 1 1 1 1 1 1 1 1 1 1 ...
 $ Time : Factor w/ 3 levels "3dpf","4dpf",..: 1 1 1 1 1 1 1 1 1 1 ...
 $ Time.numeric.: int 3 3 3 3 3 3 3 3 3 3 ...
 $ Data : num 85.73 5.87 3.47 124.27 144.13 ...

### Multi level linear modelling with clutch as a random factor

For the same reasons stated above multi-level modelling was used with clutch as a random factor.

lme1<-lmer(Data~1 + (1|Clutch), data=data, na.action=na.omit)
lme2<-update(lme1, .~. +Time)
lme3<-update(lme2, .~. +Factor1)
lme4<-update(lme3, .~. +Factor1*Time)
anova(lme1,lme2,lme3,lme4)

Data: data
Models:
lme1: Data ~ 1 + (1 | Clutch)
lme2: Data ~ (1 | Clutch) + Time
lme3: Data ~ (1 | Clutch) + Time + Factor1
lme4: Data ~ (1 | Clutch) + Time + Factor1 + Time:Factor1
 Df AIC BIC logLik deviance Chisq Chi Df
lme1 3 5239.8 5252.0 -2616.9 5233.8
lme2 5 5112.3 5132.6 -2551.1 5102.3 131.5658 2
lme3 6 5061.5 5085.9 -2524.8 5049.5 52.7555 1
lme4 8 5058.2 5090.6 -2521.1 5042.2 7.3499 2
 Pr(>Chisq)
lme1
lme2 < 0.00000000000000022 ***
lme3 0.0000000000003778 ***
lme4 0.02535 *
---
Signif. codes: 0 '***' 0.001 '**' 0.01 '*' 0.05 '.' 0.1 ' ' 1

### Assumption check

There is substantial departure from normality of the residuals, as well as, a relationship between the fitted value and residual spread and, therefore, a lack homoskedasticity. Hence, there is sufficient evidence that the assumptions of the model have been violated, therefore, shrinking transformations were attempted.

plot(lme4,col=data$Factor1,pch=as.numeric(data$Clutch)+14,cex=0.6)


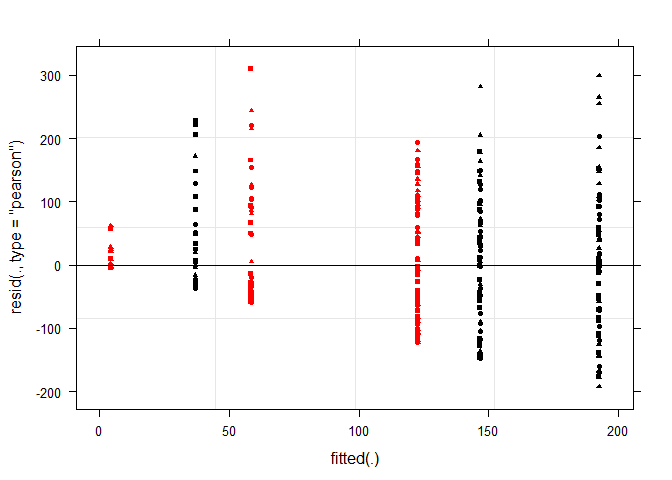


qqnorm(resid(lme2))


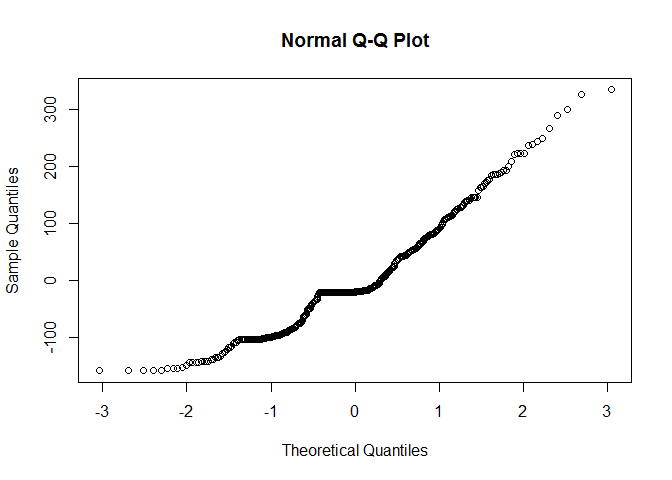


augDat <- data.frame(data,resid=residuals(lme4,type="pearson"),
 fitted=fitted(lme4))
ggplot(augDat,aes(x=Factor1,y=resid,col=Clutch))+geom_point()+geom_boxplot(aes(group=Factor1),alpha = 0.1)+coord_flip()


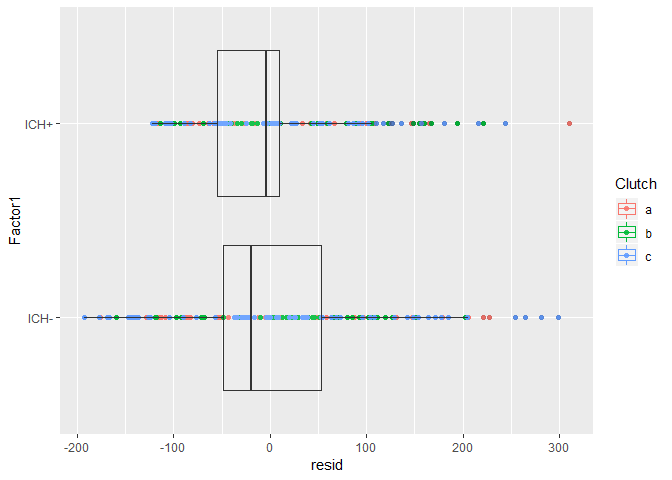


### Transformation of the data prior MLM

Several shrinking transformations were attempted and none were found to be optimal.

lme1<-lmer(log(Data+1)~1 + (1|Clutch), data=data, na.action=na.omit)
lme2<-update(lme1, .~. +Time)
lme3<-update(lme2, .~. +Factor1)
lme4<-update(lme3, .~. +Factor1*Time)
anova(lme1,lme2,lme3,lme4)

Data: data
Models:
lme1: log(Data + 1) ~ 1 + (1 | Clutch)
lme2: log(Data + 1) ~ (1 | Clutch) + Time
lme3: log(Data + 1) ~ (1 | Clutch) + Time + Factor1
lme4: log(Data + 1) ~ (1 | Clutch) + Time + Factor1 + Time:Factor1
 Df AIC BIC logLik deviance Chisq Chi Df
lme1 3 1881.2 1893.3 -937.58 1875.2
lme2 5 1675.5 1695.8 -832.75 1665.5 209.6654 2
lme3 6 1610.9 1635.2 -799.44 1598.9 66.6236 1
lme4 8 1605.6 1638.1 -794.81 1589.6 9.2557 2
 Pr(>Chisq)
lme1
lme2 < 0.00000000000000022 ***
lme3 0.0000000000000003286 ***
lme4 0.009776 **
---
Signif. codes: 0 '***' 0.001 '**' 0.01 '*' 0.05 '.' 0.1 ' ' 1

### Assumption check

No transformation appeared to sufficiently deal with the assumption violation issues. This seems to be due to the large number of zero values in the data. Therefore, a generalized mixed modelling approach was attempted.

plot(lme2,col=data$Factor1,pch=as.numeric(data$Clutch)+14,cex=0.6)


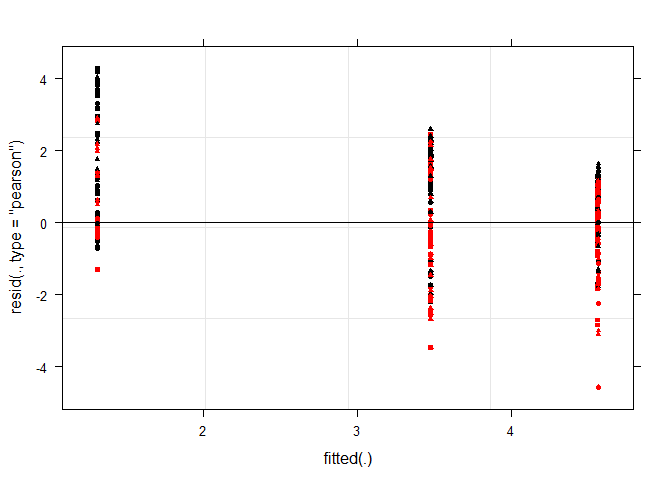


qqnorm(resid(lme2))


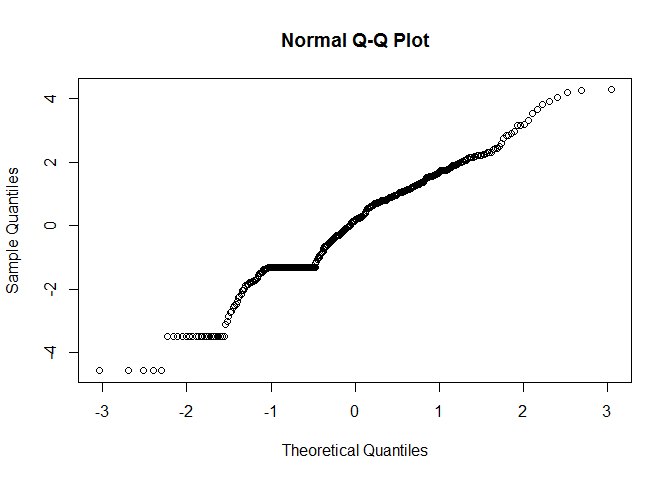


augDat <- data.frame(data,resid=residuals(lme2,type="pearson"),
 fitted=fitted(lme2))
ggplot(augDat,aes(x=Factor1,y=resid,col=Clutch))+geom_point()+geom_boxplot(aes(group=Factor1),alpha = 0.1)+coord_flip()


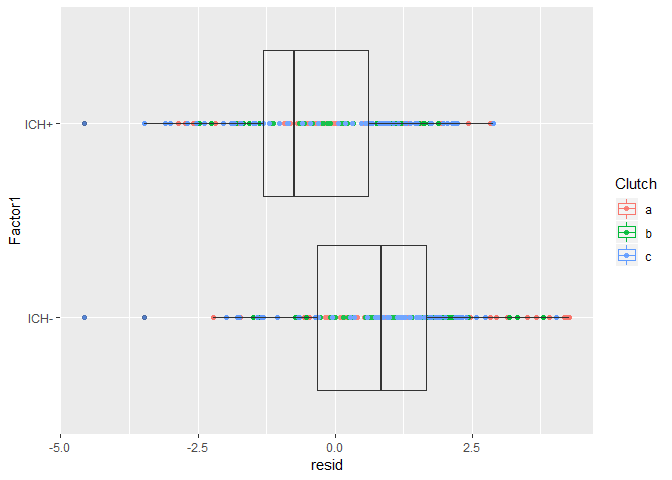


ggplot(augDat,aes(x=Time,y=resid,col=Clutch))+geom_point()+geom_boxplot(aes(group=Time),alpha = 0.1)+coord_flip()


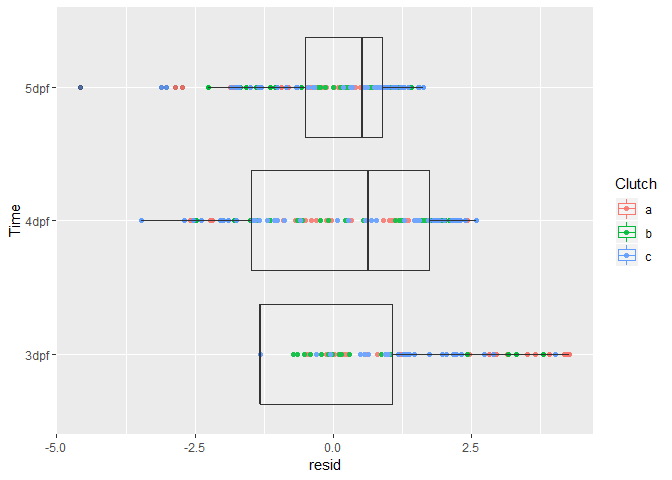


### Generalized mixed modelling

Gamma and negative binomial models can deal with low levels of zeros as they allow skewing of the data and zero inflation (for the negative binomial models only). Using the AIC values to determine the optimal model a negative binomial model with time as a factor was found to be the preferred model.

try(glmm.a<-glmmadmb(round(Data)~Factor1*Time+ (1|Clutch), data=data,family="nbinom",zeroInflation=F))

try(glmm.b<-glmmadmb(round(Data)~Factor1*Time+ (1|Clutch), data=data,family="nbinom1",zeroInflation=F))

try(glmm.c<-glmmadmb(round(Data)~Factor1*Time+ (1|Clutch), data=data,family="nbinom",zeroInflation=TRUE))

try(glmm.d<-glmmadmb(round(Data)~Factor1*Time+ (1|Clutch), data=data,family="nbinom1",zeroInflation=TRUE))

try(glmm.e<-glmer(Data+0.1~Factor1*Time+ (1|Clutch), data=data,family="Gamma"))

lme4<-lmer(round(Data)~Factor1*Time+ (1|Clutch),data=data)

try(glmm.a.time<-glmmadmb(round(Data)~Factor1*Time.numeric. +(1|Clutch), data=data,family="nbinom",zeroInflation=F))

try(glmm.b.time<-glmmadmb(round(Data)~Factor1*Time.numeric.+ (1|Clutch), data=data,family="nbinom1",zeroInflation=F))

try(glmm.c.time<-glmmadmb(round(Data)~Factor1*Time.numeric.+ (1|Clutch), data=data,family="nbinom",zeroInflation=TRUE))

try(glmm.d.time<-glmmadmb(round(Data)~Factor1*Time.numeric.+ (1|Clutch), data=data,family="nbinom1",zeroInflation=TRUE))

try(glmm.e.time<-glmer(Data+0.1~Factor1*Time.numeric.+ (1|Clutch), data=data,family="Gamma"))

lme4.time<-lmer(round(Data)~Factor1*Time.numeric.+ (1|Clutch),data=data)

AIC(glmm.a,glmm.b,lme4,glmm.a.time,glmm.b.time,glmm.c.time,glmm.d.time,lme4.time,glmm.e,glmm.e.time)

df AIC
glmm.a 8 4172.760
glmm.b 8 4027.060
lme4 8 5018.996
glmm.a.time 6 4193.640
glmm.b.time 6 4041.060
glmm.c.time 7 4177.900
glmm.d.time 7 4022.180
lme4.time 6 5038.040
glmm.e 8 4075.936
glmm.e.time 6 4141.477

### GLMM analysis

There was a significant effect of ICH, Time and a significant interaction between the two. This suggests ICH fish are significantly less mobile and how their mobility changes of time is significantly effected.

glmm1<-glmmadmb(round(Data)~1 + (1|Clutch), data=data,family="nbinom1")
glmm2<-update(glmm1, .~. +Time)
glmm3<-update(glmm2, .~. +Factor1)
glmm4<-update(glmm3, .~. +Factor1*Time)
anova(glmm1,glmm2,glmm3,glmm4)

Analysis of Deviance Table

Model 1: round(Data) ~ 1
Model 2: round(Data) ~ +Time
Model 3: round(Data) ~ +Time + Factor1
Model 4: round(Data) ~ +Time + Factor1 + Time:Factor1
 NoPar LogLik Df Deviance Pr(>Chi)
1 3 -2155.6
2 5 -2044.3 2 222.52 < 0.00000000000000022 ***
3 6 -2011.1 1 66.38 0.0000000000000003331 ***
4 8 -2005.5 2 11.22 0.003661 **
---
Signif. codes: 0 '***' 0.001 '**' 0.01 '*' 0.05 '.' 0.1 ' ' 1

### Assumption check

The residuals appeared to center around zero at each factor level and follow an approximate negative binomial distribution. Therefore, there is little evidence to suggest the assumptions of the model have been violated.

augDat <- data.frame(data,resid=residuals(glmm4,type="pearson"),
 fitted=fitted(glmm4))
ggplot(augDat,aes(x=Factor1,y=resid,col=Clutch))+geom_point()+geom_boxplot(aes(group=Factor1),alpha = 0.1)+coord_flip()


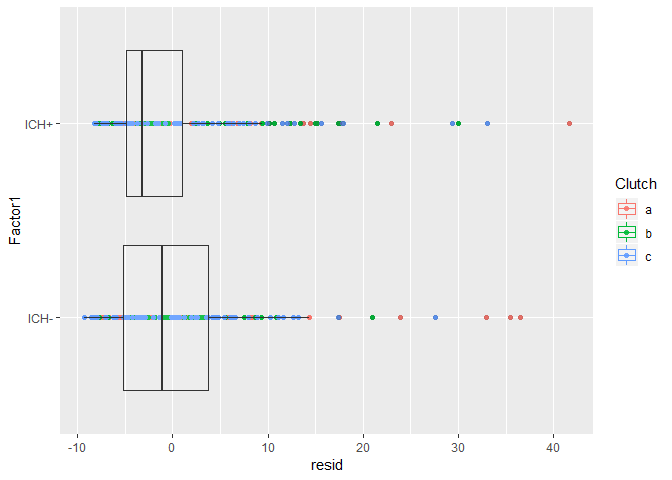


ggplot(augDat,aes(x=Time,y=resid,col=Factor1))+geom_point()+geom_boxplot(aes(group=Time),alpha = 0.1)+coord_flip()


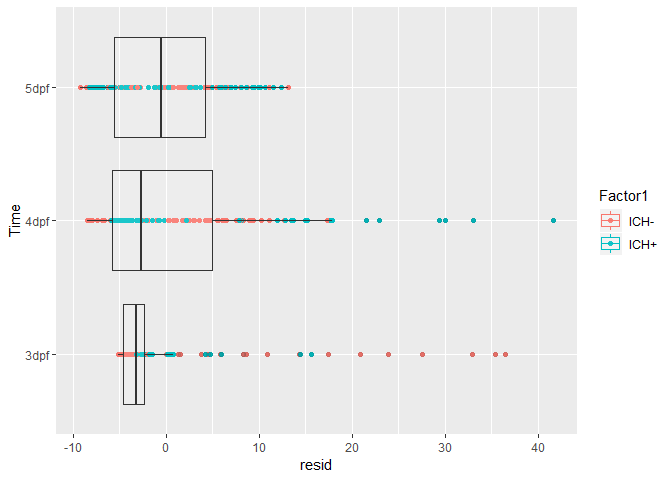


### Post-hoc analyses

Post-hoc analyses reveal that ICH significantly reduces movement at day 3 and day 4, and approaches significance at day 5.

post <- lsmeans(glmm4, pairwise ~ Factor1*Time, adjust="none")
posthoc<-summary(post)


comparisons<-c(1,10,15)

p.value<-data.frame(cbind(posthoc$contrasts["contrast"],posthoc$contrasts["p.value"]))

p<-p.value[comparisons,2]
comp<-p.value[comparisons,]
padj<-p.adjust(p, method="holm", n= length(p))
ptable<-data.frame(cbind(comp,padj))
print(ptable, row.names=FALSE)

contrast p.value padj
 ICH-,3dpf - ICH+,3dpf 0.00002112242 0.00006336726
 ICH-,4dpf - ICH+,4dpf 0.00158469930 0.00316939859
 ICH-,5dpf - ICH+,5dpf 0.08225743993 0.08225743993

### Logistic regression of mobility

Because of the non-movers a logistic regression was performed comparing the proportions of non-movers and movers between groups at each time-point. From this a very similar picture emerges with significant effects of ICH at day 3 and day 4, but not day 5.

data$move<-1*(data$Data>0)

glmm1<-glmer(move~1 + (1|Clutch), data=data,family="binomial")
glmm2<-update(glmm1, .~. +Time)
glmm3<-update(glmm2, .~. +Factor1)
glmm4<-update(glmm3, .~. +Factor1*Time)
anova(glmm1,glmm2,glmm3,glmm4)

Data: data
Models:
glmm1: move ~ 1 + (1 | Clutch)
glmm2: move ~ (1 | Clutch) + Time
glmm3: move ~ (1 | Clutch) + Time + Factor1
glmm4: move ~ (1 | Clutch) + Time + Factor1 + Time:Factor1
 Df AIC BIC logLik deviance Chisq Chi Df
glmm1 2 462.06 470.18 -229.03 458.06
glmm2 4 364.97 381.21 -178.49 356.97 101.0889 2
glmm3 5 339.49 359.78 -164.74 329.49 27.4852 1
glmm4 7 341.40 369.82 -163.70 327.40 2.0848 2
 Pr(>Chisq)
glmm1
glmm2 < 0.00000000000000022 ***
glmm3 0.0000001583 ***
glmm4 0.3526
---
Signif. codes: 0 '***' 0.001 '**' 0.01 '*' 0.05 '.' 0.1 ' ' 1

post <- lsmeans(glmm4, pairwise ~ Factor1*Time, adjust="none")
posthoc<-summary(post)


comparisons<-c(1,10,15)

p.value<-data.frame(cbind(posthoc$contrasts["contrast"],posthoc$contrasts["p.value"]))

p<-p.value[comparisons,2]
comp<-p.value[comparisons,]
padj<-p.adjust(p, method="holm", n= length(p))
ptable<-data.frame(cbind(comp,padj))
print(ptable, row.names=FALSE)

contrast p.value padj
 ICH-,3dpf - ICH+,3dpf 0.0001276988 0.0003830965
 ICH-,4dpf - ICH+,4dpf 0.0016640427 0.0033280854
 ICH-,5dpf - ICH+,5dpf 0.6507306563 0.6507306563

## BBH macrophage counts

### Loading data

data<-read.csv("leukocytemacs.csv", header=T)
str(data)

'data.frame': 36 obs. of 3 variables:
 $ Clutch : Factor w/ 2 levels "a","b": 1 1 1 1 1 1 1 1 1 1 ...
 $ Factor1: Factor w/ 2 levels "ICH-","ICH+": 1 1 1 1 1 1 1 1 1 1 ...
 $ Data : int 18 16 4 11 4 11 8 19 2 3 ...

### Generalized mixed modelling

Because this is count data Poisson and negative binomial models were attempted. Using the AIC values to determine the optimal model, a negative binomial model was selected. The negative binomial performed better most likely due to over-dispression of the data.

try(glmm.a<-glmmadmb(Data~Factor1+ (1|Clutch), data=data,family="nbinom",zeroInflation=F))

try(glmm.b<-glmmadmb(Data~Factor1+ (1|Clutch), data=data,family="nbinom1",zeroInflation=F))

try(glmm.c<-glmer(Data~Factor1+ (1|Clutch), data=data, family=poisson(link="log")))

try(glmm.d<-glmer(Data~Factor1+ (1|Clutch), data=data, family=poisson(link="identity")))

try(glmm.e<-glmer(Data~Factor1+ (1|Clutch), data=data,family=poisson(link="sqrt")))

AIC(glmm.a,glmm.b,glmm.c,glmm.d,glmm.e)

df AIC
glmm.a 4 270.5040
glmm.b 4 273.9180
glmm.c 3 446.7101
glmm.d 3 475.2077
glmm.e 3 461.6097

### GLMM effecst of ICH

The log-likelihood test reveals that including ICH into the model does significantly improve the model. The inference of this is that ICH does significantly effect macrophages numbers.

glmm1<-glmmadmb(round(Data)~1 + (1|Clutch), data=data,family="nbinom")
glmm2<-update(glmm1, .~. +Factor1)
anova(glmm1,glmm2)

Analysis of Deviance Table

Model 1: round(Data) ~ 1
Model 2: round(Data) ~ +Factor1
 NoPar LogLik Df Deviance Pr(>Chi)
1 3 -134.27
2 4 -131.25 1 6.028 0.01408 *
---
Signif. codes: 0 '***' 0.001 '**' 0.01 '*' 0.05 '.' 0.1 ' ' 1

summary(glmm2)

Call:
glmmadmb(formula = round(Data) ~ (1 | Clutch) + Factor1, data = data,
 family = "nbinom")

AIC: 270.5

Coefficients:
 Estimate Std. Error z value Pr(>|z|)
(Intercept) 2.261 0.230 9.82 <0.0000000000000002 ***
Factor1ICH+ 0.733 0.289 2.54 0.011 *
---
Signif. codes: 0 '***' 0.001 '**' 0.01 '*' 0.05 '.' 0.1 ' ' 1

Number of observations: total=36, Clutch=2
Random effect variance(s):
Group=Clutch
 Variance StdDev
(Intercept) 0.02633 0.1623

Negative binomial dispersion parameter: 1.6414 (std. err.: 0.45703)

Log-likelihood: -131.252

### Assumption check

The residuals appeared to center around zero at each factor level and follow an approximate negative binomial distribution. Therefore, there is little evidence to suggest the assumptions of the model have been violated.

augDat <- data.frame(data,resid=residuals(glmm2,type="pearson"),
 fitted=fitted(glmm2))
ggplot(augDat,aes(x=Factor1,y=resid,col=Clutch))+geom_point()+geom_boxplot(aes(group=Factor1),alpha = 0.1)+coord_flip()


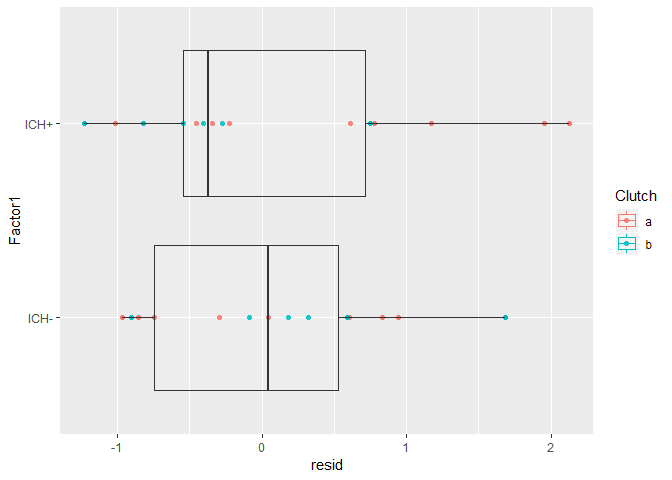


## BBH neutrophil counts

### Loading data

data<-read.csv("leukocyteneuts.csv", header=T)
str(data)

'data.frame': 33 obs. of 3 variables:
 $ Clutch : Factor w/ 2 levels "a","b": 1 1 1 1 1 1 1 1 1 1 ...
 $ Factor1: Factor w/ 2 levels "ICH-","ICH+": 1 1 1 1 1 1 1 1 1 1 ...
 $ Data : int 15 23 16 16 18 18 29 22 22 5 ...

### Generalized mixed modelling

Because this is count data Poisson and negative binomial models were attempted. Using the AIC values to determine the optimal model, a negative binomial model was selected. The negative binomial performed better most likely due to over-dispression of the data.

try(glmm.a<-glmmadmb(Data~Factor1+ (1|Clutch), data=data,family="nbinom",zeroInflation=F))

try(glmm.b<-glmmadmb(Data~Factor1+ (1|Clutch), data=data,family="nbinom1",zeroInflation=F))

try(glmm.c<-glmer(Data~Factor1+ (1|Clutch), data=data, family=poisson(link="log")))

try(glmm.d<-glmer(Data~Factor1+ (1|Clutch), data=data, family=poisson(link="identity")))

try(glmm.e<-glmer(Data~Factor1+ (1|Clutch), data=data,family=poisson(link="sqrt")))

AIC(glmm.a,glmm.b,glmm.c,glmm.d,glmm.e)

df AIC
glmm.a 4 242.8800
glmm.b 4 242.5840
glmm.c 3 284.7307
glmm.d 3 286.2954
glmm.e 3 285.5298

### GLMM effecst of ICH

The log-likelihood test reveals that including ICH into the model does not significantly improve the model. The inference of this is that ICH does not significantly effect neutrophil numbers.

glmm1<-glmmadmb(round(Data)~1 + (1|Clutch), data=data,family="nbinom1")
glmm2<-update(glmm1, .~. +Factor1)
anova(glmm1,glmm2)

Analysis of Deviance Table

Model 1: round(Data) ~ 1
Model 2: round(Data) ~ +Factor1
 NoPar LogLik Df Deviance Pr(>Chi)
1 3 -117.50
2 4 -117.29 1 0.414 0.5199

### Assumption check

The residuals appeared to center around zero at each factor level and follow an approximate negative binomial distribution. Therefore, there is little evidence to suggest the assumptions of the model have been violated.

augDat <- data.frame(data,resid=residuals(glmm2,type="pearson"),
 fitted=fitted(glmm2))
ggplot(augDat,aes(x=Factor1,y=resid,col=Clutch))+geom_point()+geom_boxplot(aes(group=Factor1),alpha = 0.1)+coord_flip()


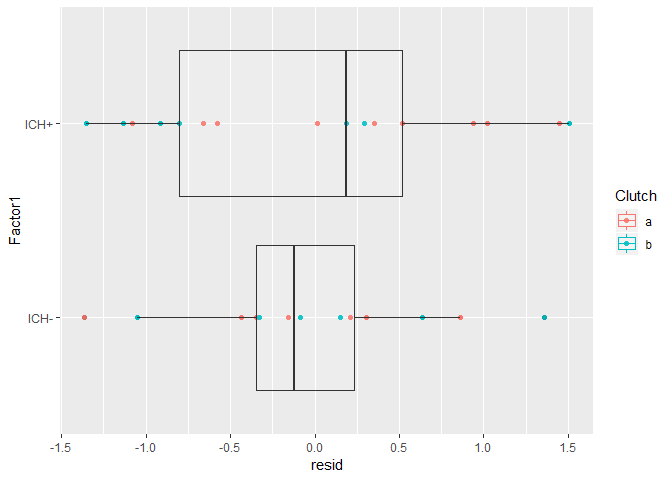

Supplement: Supplementary file 4 [file f1000research-7-18516-s0003.tgz › 32bba787-aad6-401a-9809-73c53c9d765f_Sup_file_3.docx]
