# Supplementary figures and images for: Using zebrafish larval models to study brain injury, locomotor and neuroinflammatory outcomes following intracerebral haemorrhage
Source: F1000Res. 2018 Nov 8;7:1617. Originally published 2018 Oct 8. [Version 2] doi: 10.12688/f1000research.16473.2 (PMC6234746; doi:10.12688/f1000research.16473.2)

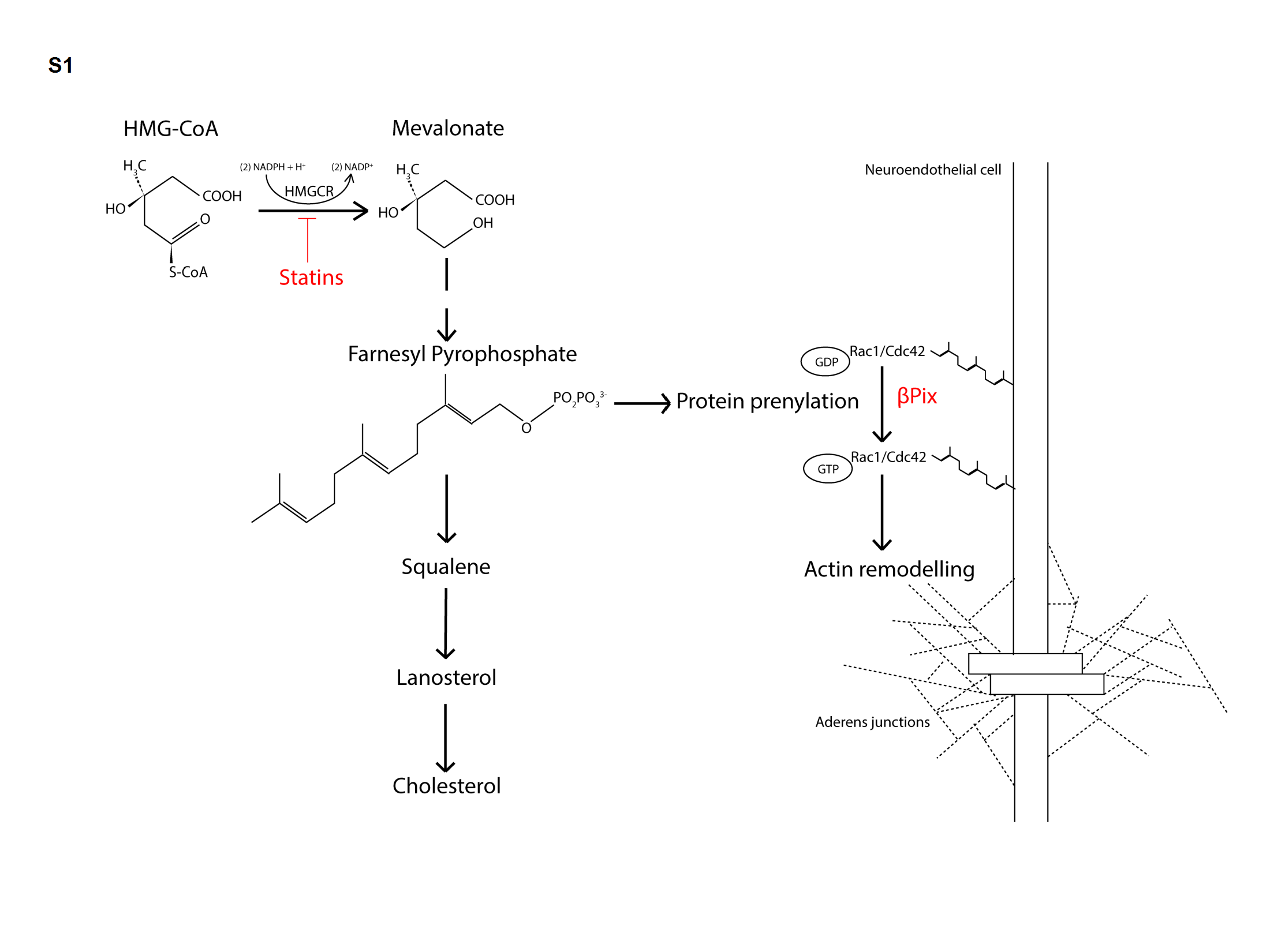

Supplement: Supplementary file 5 [file f1000research-7-18516-s0004.tgz › 7d3a670e-1f0c-41e3-9310-bf04c359b483_Supplementary_Figure_1.tiff]

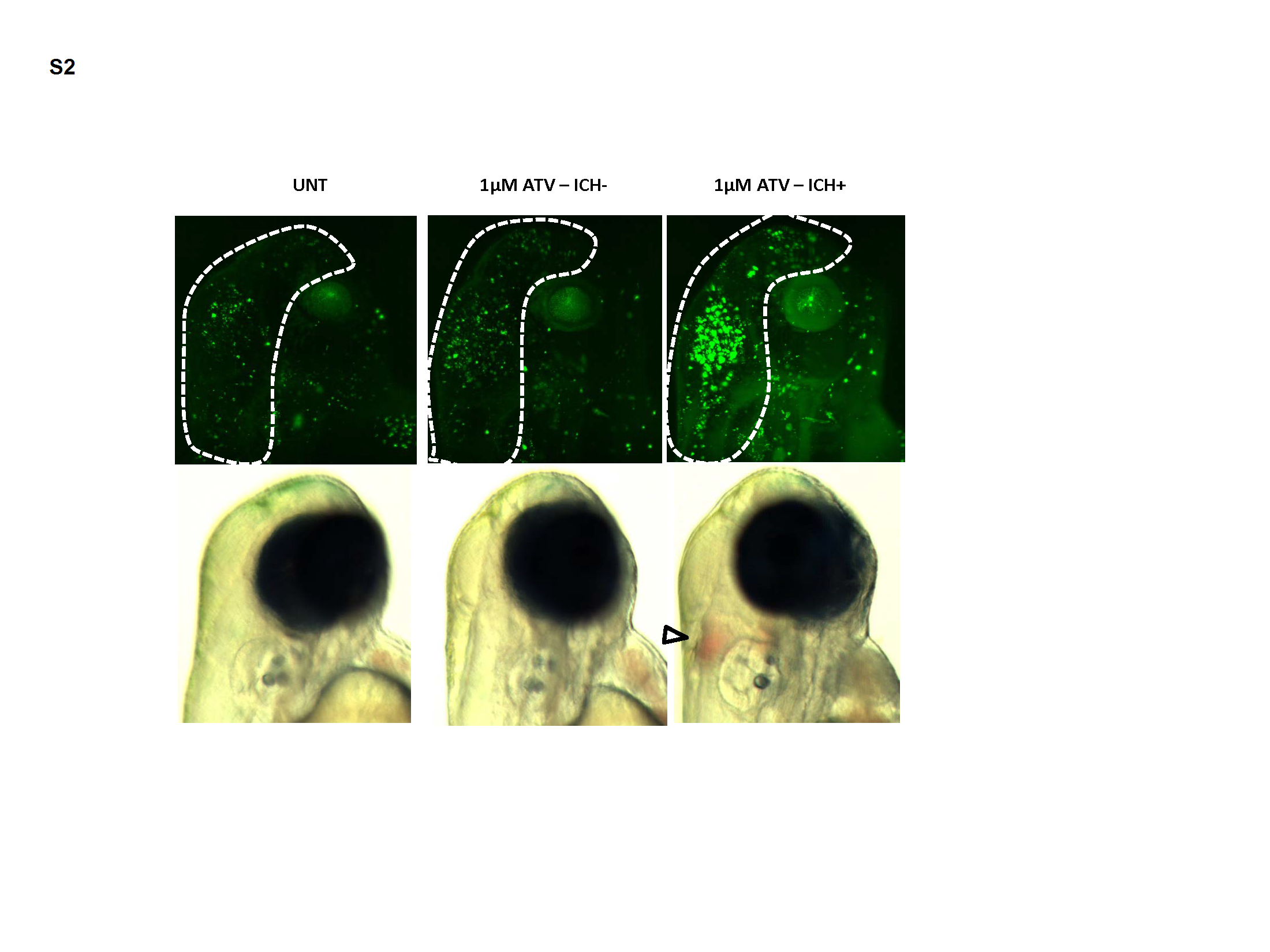

Supplement: Supplementary file 6 [file f1000research-7-18516-s0005.tgz › 63988fa1-896c-4e57-b00e-3b4768bc311f_Supplementary_Figure_2.tif]

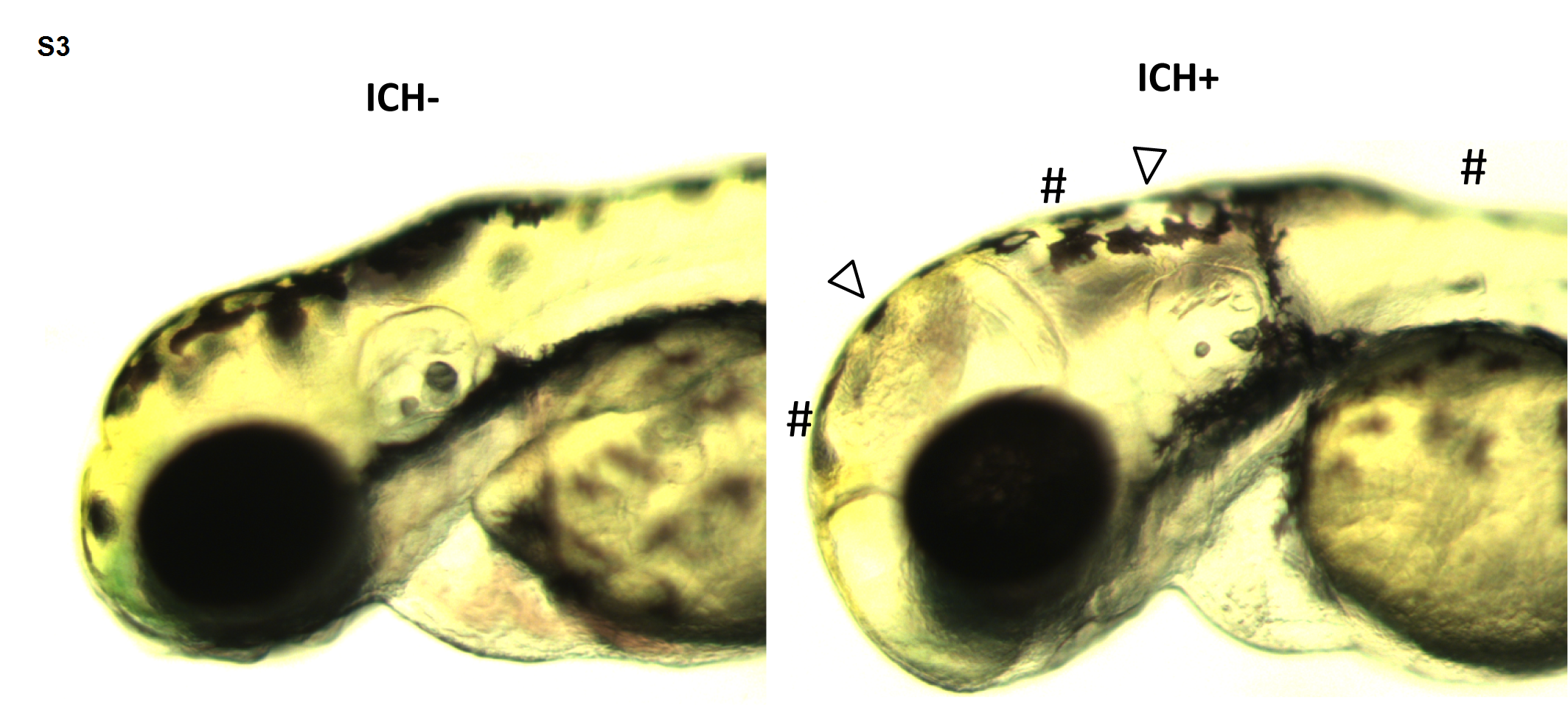

Supplement: Supplementary file 7 [file f1000research-7-18516-s0006.tgz › 7c247bb3-2896-400e-9fe8-8704170a4fb3_Supplementary_Figure_3.tif]

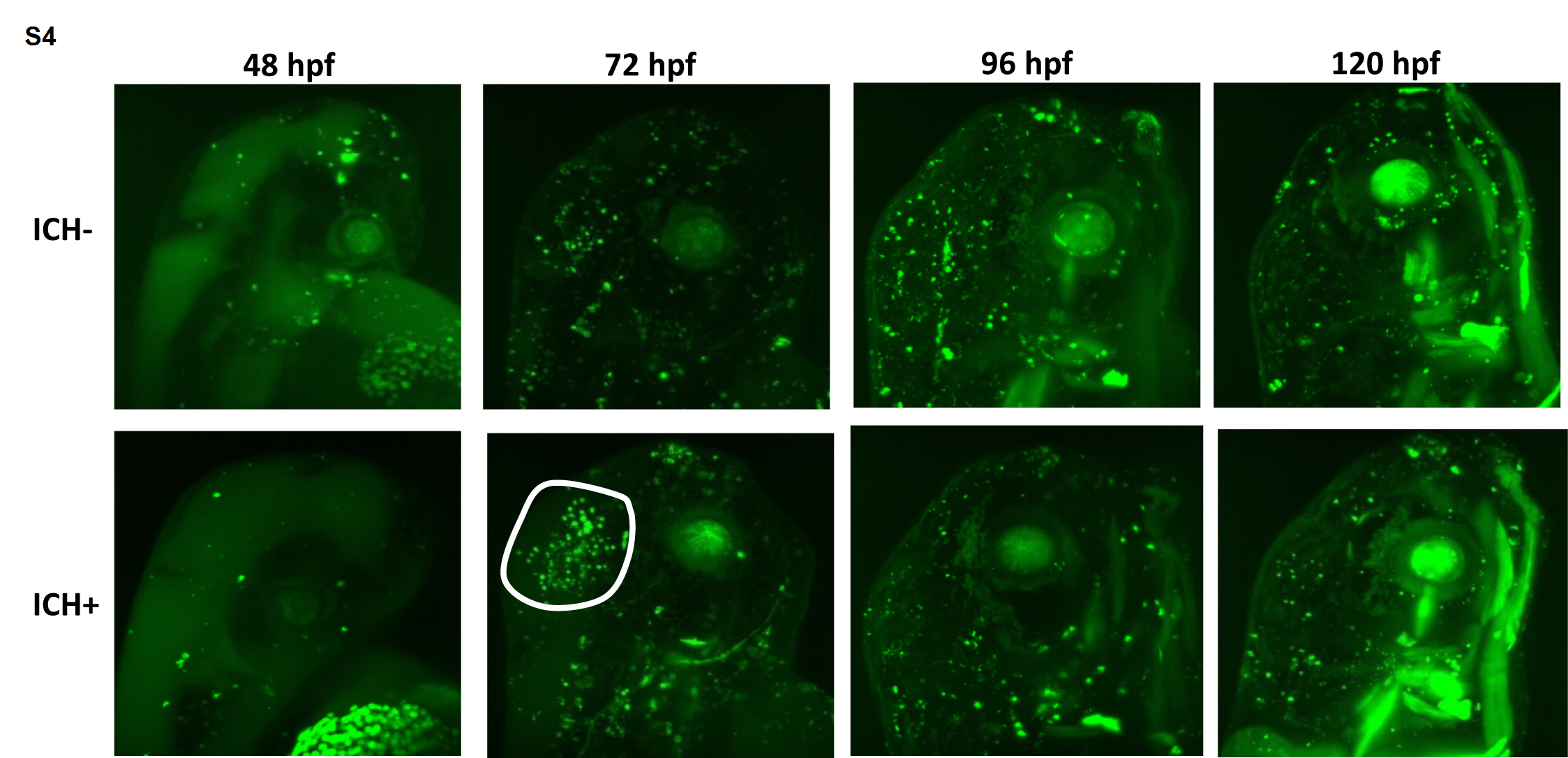

Supplement: Supplementary file 8 [file f1000research-7-18516-s0007.tgz › c5ce58dd-020e-4afd-8cd5-52d27b2fe761_Supplementary_Figure_4.tif]

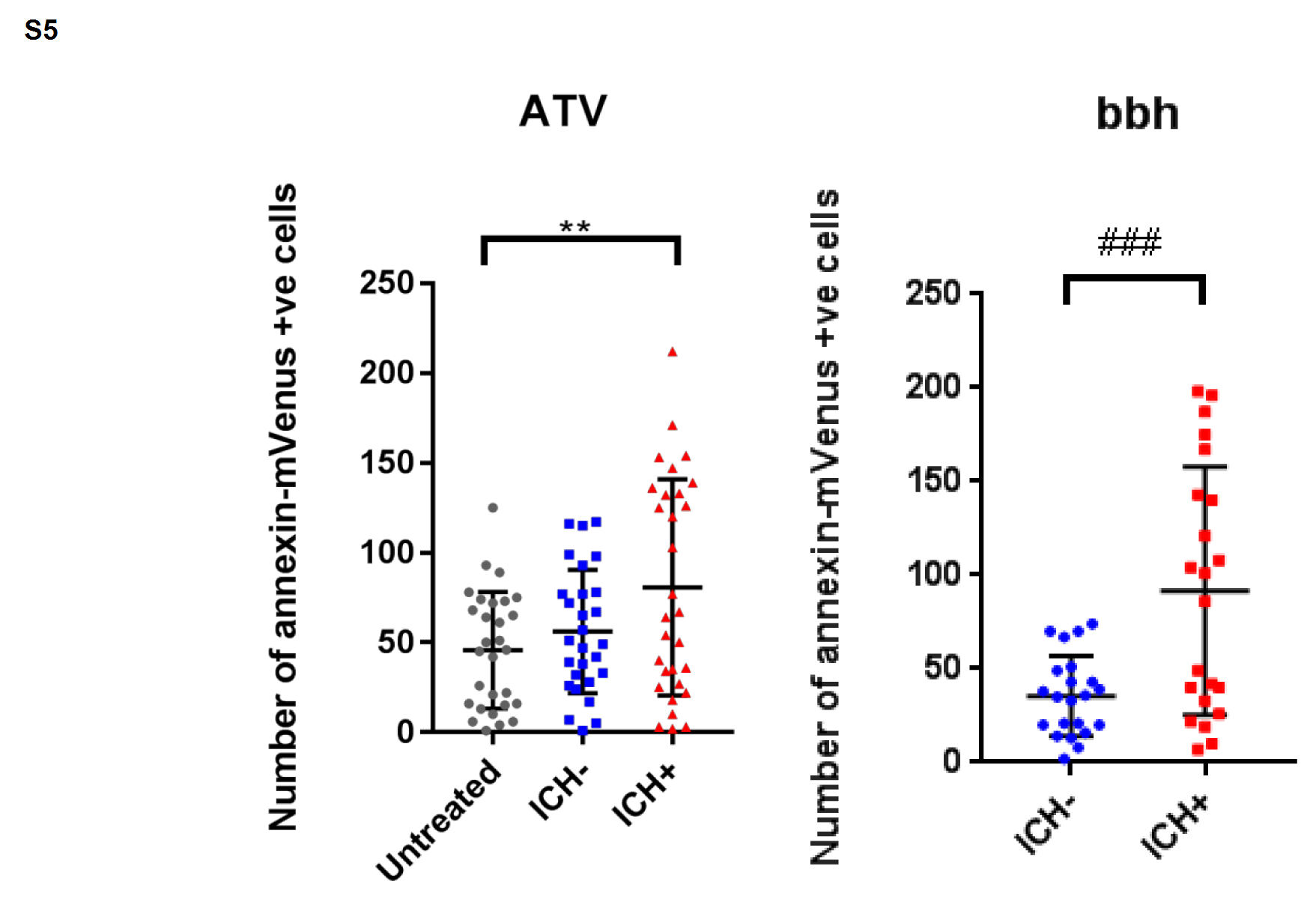

Supplement: Supplementary file 9 [file f1000research-7-18516-s0008.tgz › 505af6a9-d450-44f1-ad1c-363bcceea3b7_Supplementary_Figure_5.tif]
