# Supplementary material for: Using zebrafish larval models to study brain injury, locomotor and neuroinflammatory outcomes following intracerebral haemorrhage
Source: F1000Res. 2018 Nov 8;7:1617. Originally published 2018 Oct 8. [Version 2] doi: 10.12688/f1000research.16473.2 (PMC6234746; doi:10.12688/f1000research.16473.2)
Supplement: Peer review history

# Peer review: Reviewer response for version 2

**DOI:** 10.5256/f1000research.18516.r40436

**Published:** 2018-11-12

**Authors:** Marietta Zille

**Affiliations:** 1 University of Lübeck, Institute for Experimental and Clinical Pharmacology and Toxicology, Lübeck, Germany; 2 Fraunhofer Research Institution for Marine Biotechnology and Cell Technology, Lübeck, Germany

I would like to thank the authors for carefully considering the reviewers’ comments. I have no further comments and look forward to future studies using this zebrafish ICH model.

Is the rationale for developing the new method (or application) clearly explained?

Yes

Is the description of the method technically sound?

Yes

Are the conclusions about the method and its performance adequately supported by the findings presented in the article?

Yes

If any results are presented, are all the source data underlying the results available to ensure full reproducibility?

Yes

Are sufficient details provided to allow replication of the method development and its use by others?

Yes

Reviewer Expertise:

NA

I confirm that I have read this submission and believe that I have an appropriate level of expertise to confirm that it is of an acceptable scientific standard.

# Peer review: Reviewer response for version 1

**DOI:** 10.5256/f1000research.18006.r39224

**Published:** 2018-10-30

**Authors:** Marietta Zille

**Affiliations:** 1 University of Lübeck, Institute for Experimental and Clinical Pharmacology and Toxicology, Lübeck, Germany; 2 Fraunhofer Research Institution for Marine Biotechnology and Cell Technology, Lübeck, Germany

In this manuscript, the authors propose two models of intracerebral hemorrhage (ICH) in zebrafish larvae to study post-hemorrhage brain injury. The authors compare a genetic (bubblehead) and chemically inducible (atorvastatin) model of spontaneous ICH and elegantly show assessment of cell death and inflammation in these models using live non-invasive imaging. In addition, they present data on locomotor activity as a functional outcome measurement.

 First of all, I want to congratulate the authors on this high quality study that is timely and relevant to the ICH community as spontaneous models that allow higher throughput screening of potentially protective or regenerative compounds are clearly needed in the field ( Hemorrhagic Stroke Academia Industry (HEADS) Roundtable Participants, 2018). Although new to the ICH community, zebrafish models have multiple advantages that may be of great use in this research area (as well as in others).

 I have a couple of suggestions that I hope will be beneficial to further improve the quality of this excellent publication:

1. We and others have recently established that neurons die by multiple different regulated cell death mechanisms after ICH, including ferroptosis and necroptosis ( Zille et al, 2017, Li et al, 2018). Therefore, the authors should discuss the limitation of the cell death marker, Annexin V, used in their study. Annexin V detects exposure of phosphatidiyl serine on the cell surface. On the one hand, this is not specific to apoptosis since when the cell membrane is disrupted phosphatidylserine can be detected intracellularly (and hence should not be claimed to analyze apoptotic cell death, e.g. in Methods section) ( Munoz et al, 2013, Sawai et al, 2011, Zille et al, 2012). On the other hand, this only reflects a specific subset of cell death. This may also explain why cell death was detected only in a subset of larvae and at one specific time point which is also a relatively narrow time window. Is there a possibility to determine the involvement of other cell death mechanisms in zebrafish (e.g., GPX4 for ferroptosis or pRIP1/3 for necroptosis)? Undoubtedly, the exact cell death mechanisms warrant further exploration in future studies including pharmacological and molecular interventions.
2. The authors nicely show side-by-side comparison of both bubblehead and atorvastatin model. What is the main difference between the two? Therefore, the authors should make fluorescence pictures from both available in Fig. 2, the same time course in Fig. 3 as well as subgroup analysis in Fig. 4 and 5. Was only one of the two investigated regarding macrophages or are they pooled in this analysis?
3. One of the differences seems to be the occurrence of edema in the bubblehead model. This is very interesting as it is also a key feature in the ICH pathophysiology. The authors should provide pictures on both models over time. Is it possible to quantify the edema?
4. Locomotor activity: How does swimming time represent functional outcome of the larvae? Would distance travelled be an alternative readout?
5. What is the size of the hematoma and does it correlate with cell death, locomotor activity or macrophage activation?
6. For the atorvastatin model, was the 1µM dose used in Figure 2-5? If not, maybe the clusters of positive and negative for Annexin V correspond to the dose of atorvastatin?
7. One of the major disadvantages of the larvae model is that it uses young animals. Is it possible to use atorvastatin to induce ICH in old zebrafish?
8. Regarding modeling human disease, another difference is that there is no mortality in this model at least not to the time points investigated. Do they die at earlier time points than their wildtype/untreated littermates? Do higher dosages of atorvastatin induce mortality in the larvae due to ICH?

Minor:

1.  From the data where replicates were acquired, please specify whether the mean or median was chosen.
2. What is the % MS222 used as lethal overdose?

**Are a suitable application and appropriate end-users identified?**

 Yes

**If applicable, is the statistical analysis and its interpretation appropriate?**

 Yes

**Are the 3Rs implications of the work described accurately?**

 Yes

Is the rationale for developing the new method (or application) clearly explained?

Yes

Is the description of the method technically sound?

Yes

Are the conclusions about the method and its performance adequately supported by the findings presented in the article?

Yes

If any results are presented, are all the source data underlying the results available to ensure full reproducibility?

Yes

Are sufficient details provided to allow replication of the method development and its use by others?

Yes

Reviewer Expertise:

Intracerebral haemorrhage, stroke researcher, cell death mechanisms

I confirm that I have read this submission and believe that I have an appropriate level of expertise to confirm that it is of an acceptable scientific standard, however I have significant reservations, as outlined above.

# Response

**Published:** 2018-11-02

**Authors:** siobhan crilly

**Affiliations:** The University of Manchester, UK

Dear Dr Zille,

 Many thanks indeed for reviewing our manuscript and your helpful comments. Please see below for responses to your specific questions. Where necessary, we have made amendments in a second version of our manuscript, which we also refer to below.

***We and others have recently established that neurons die by multiple different regulated cell death mechanisms after ICH, including ferroptosis and necroptosis (*** ***Zille et al, 2017*** ***,*** ***Li et al, 2018*** ***). Therefore, the authors should discuss the limitation of the cell death marker, Annexin V, used in their study. Annexin V detects exposure of phosphatidiyl serine on the cell surface. On the one hand, this is not specific to apoptosis since when the cell membrane is disrupted phosphatidylserine can be detected intracellularly (and hence should not be claimed to analyze apoptotic cell death, e.g. in Methods section) (*** ***Munoz et al, 2013*** ***,*** ***Sawai et al, 2011*** ***,*** ***Zille et al, 2012*** ***). On the other hand, this only reflects a specific subset of cell death. This may also explain why cell death was detected only in a subset of larvae and at one specific time point which is also a relatively narrow time window. Is there a possibility to determine the involvement of other cell death mechanisms in zebrafish (e.g., GPX4 for ferroptosis or pRIP1/3 for necroptosis)? Undoubtedly, the exact cell death mechanisms warrant further exploration in future studies including pharmacological and molecular interventions.***

 We thank the reviewer for raising this point, and we appreciate that only using Annexin V in this study limits the information that we can obtain about cell death mechanisms. As suggested, we have updated our discussion in version 2 to address this, and removed the term ‘apoptosis’ throughout. Of interest, in addition to the Annexin V reporter line, we have also verified cell death using an acridine orange assay in live larvae at the same time points and found an identical pattern of brain lesions in haemorrhaged animals only at 72 hpf (data not shown). The primary purpose of this paper was to demonstrate that a range of ‘translatable’ disease outcomes exist following ICH in zebrafish larvae. However, our ongoing studies will aim to build a more accurate understanding of the precise mechanisms underlying brain injury following ICH in zebrafish larvae, which will include an investigation into other forms of cell death, such as necroptosis and ferroptosis.

***The authors nicely show side-by-side comparison of both bubblehead and atorvastatin model. What is the main difference between the two?***

 Exposure to the HMG-CoA reductase inhibitor, atorvastatin (ATV) at 24 hours post-fertilisation (hpf) leads to spontaneous cerebral-specific blood vessel rupture at ~33hpf in zebrafish larvae at the onset of circulation (Eisa-Beygi et al., 2013, Shen et al., 2013, Huang et al., 2017, Li et al., 2017). By inhibiting HMG-CoA in neovascular neuroendothelial cells, ATV disrupts isoprenylation and hence activation of Rac1 (Xiao et al., 2013) and subsequent actin remodelling to stabilise VE cadherin mediated tight junctions (Eisa-Beygi and Rezaei, 2016). The result of this impairment is a ‘leaky’ cerebrovasculature and ICH between 33-48hpf. Comparably, the ‘bubblehead’ (bbh) mutant line, which expresses a hypomorphic mutation in the *arhgef7* gene, encoding the Rac GEF βpix, also exhibit spontaneous ICH, as well as hydrocephalus, within a similar time frame to the ATV model (Liu et al., 2007, ten Klooster et al., 2006).  Therefore it appears ICH is induced through comparable mechanistic defects in both ATV and bbh models. We have included a summary of this difference in new supplementary figure 1 in version 2.

***Therefore, the authors should make fluorescence pictures from both available in Fig. 2, the same time course in Fig. 3 as well as subgroup analysis in Fig. 4 and 5. Was only one of the two investigated regarding macrophages or are they pooled in this analysis?***

 In our updated version, we have now included images of the ATV model with Annexin V cell death lesions at 3 dpf in supplementary figure 2. We have monitored locomotor function in the ATV model larvae over the same time course as figure 3, however the baseline measurements of control larvae at 3 and 4 dpf was too low for a measurable outcome, and so we continued with the bbh mutant. We hypothesise this could be due to individual strain effect, as we have observed variable locomotor function in normal larvae between different strains. The ATV model was not used for the studies in figure 4 and 5 due to the anti-inflammatory effects of statins (Bu et al., 2011).

***One of the differences seems to be the occurrence of edema in the bubblehead model. This is very interesting as it is also a key feature in the ICH pathophysiology. The authors should provide pictures on both models over time. Is it possible to quantify the edema?***

 For the ATV model of ICH, larvae do not exhibit any oedema at any age. Also, although oedema occurs exclusively in ICH+ bbh homozygotes it occurs in a stochastic fashion. Liu et al (2007) have shown that oedema and haemorrhage occur as separate entities in bbh mutants and develop independently of each other. For these reasons and the limitations raised in our discussion, we avoided using oedema as a pathological outcome for haemorrhagic stroke.

***Locomotor activity: How does swimming time represent functional outcome of the larvae? Would distance travelled be an alternative readout?***

 We have recorded distance travelled for both models in conjunction to time spent mobile and the trend is identical to that shown in Figure 3. We decided to use cumulative time spent mobile as this would be inclusive of seizure-like behaviour as well as normal thigmotactic behaviour (Turrini et al., 2017)

***What is the size of the hematoma and does it correlate with cell death, locomotor activity or macrophage activation?***

 This is an extremely interesting question, and will form the basis of a future grant application. We have not accurately measured haematoma size, however we will use 3D rendering and light sheet microscopy to do so in the future. We are also interested in investigating haematoma location within the brain, to determine if this effects behavioural outcomes/specific neuronal populations.

***For the atorvastatin model, was the 1µM dose used in Figure 2-5? If not, maybe the clusters of positive and negative for Annexin V correspond to the dose of atorvastatin?***

 Yes, we used 1uM ATV, however we only performed ATV experiments in Figure 1-3, as discussed in methods and in response to your earlier question. Based on observation, we do not think ATV dosage affects severity of haemorrhage or haemorrhage-induced injury; rather it just affects the frequency of haemorrhage occurrence. We propose that once ICH occurs, the pathogenic response to blood in the brain is comparable between larvae, irrespective of ATV dosage.

***One of the major disadvantages of the larvae model is that it uses young animals. Is it possible to use atorvastatin to induce ICH in old zebrafish?***

 We have used ATV to treat larvae at later time points to verify the findings in Eisa-Beygi et al (2013) and saw that haemorrhages were only induced when embryos are exposed to ATV prior to the critical neuroendothelial developmental time point  at ~36-48hpf. Animals treated after this time do not haemorrhage.

 We believe that the use of young animals is an advantage, especially given the potential for impacting the 3Rs. Furthermore as the animals age, pigmentation develops and the larvae are no longer transparent – therefore this significant experimental advantage will be lost using older animals. Admittedly, ICH is predominantly a disease associated with aging; however this condition does occur in children and young adults, making our model even more pertinent for understanding those types of presentation. Ultimately, however, our article does strongly indicate that ICH induces brain injury phenotypes in young zebrafish that are comparable to the aged human brain. Furthermore, through our observations, we know that zebrafish larvae recover from ICH at an astonishing rate and therefore offer a potentially powerful tool for studying resolution and recovery processes in the future.

***Regarding*** ***m*** ***odelling human disease, another difference is that there is no mortality in this model at least not to the time points investigated. Do they die at earlier time points than their wildtype/untreated littermates?***

 The bbh mutants do not die due to ICH. Indeed, these animals develop into healthy, fertile adults, which we propose is due to either developmental processes or the regenerate properties of zebrafish. Either way, understanding this recovery at the molecular/cellular level could provide essential insight into how we may one day recover the injured human brain.

***Do higher dosages of atorvastatin induce mortality in the larvae due to ICH?***

 In the ATV model, high doses of ATV will kill the larvae; however we propose this is due to toxicity rather than ICH-related.

***From the data where replicates were acquired, please specify whether the mean or median was chosen.***

 Mean was presented throughout. This has now been clarified in the latest version of the manuscript.

***What is the % MS222 used as lethal overdose?*** 

 4% MS222 followed by maceration or freezing, which we have made clear in version 2.

 Kind regards

 Siobhan Crilly & Paul Kasher

# Peer review: Reviewer response for version 1

**DOI:** 10.5256/f1000research.18006.r39725

**Published:** 2018-10-24

**Authors:** Claire L. Gibson

**Affiliations:** 1 University of Nottingham , Nottingham, UK

This article describes the development of a zebrafish model of intracerebral haemorrhage (ICH). ICH is a significant contributor to human mortality and morbidity and effect treatment options are limited.  Research in this area has been hindered by the lack of appropriate models – although rodent models have contributed to our understanding of the pathology underlying ICH such models are based on a surgical intervention which does not mirror the spontaneous event, in humans, of ICH. Here the authors compare an existing genetic (bubblehead) and chemically inducible (atorvastatin) zebrafish models of spontaneous ICSH and importantly assess brain injury, locomotor function and neuroinflammation.

 The article is well described with sufficient experimental detail and the authors clearly articulate the 3Rs benefits of such work. One of the particularly novel aspects of this work is the ability to image in vivo the ICH-induced inflammatory processes and cellular interactions – this is not currently feasible in whole animal models, such as rodents. This may allow improved understanding of the early innate responses to spontaneous bleeding as occurs in ICH patients but which we cannot model in rodents.

 Although the authors state that this paper used equipment and procedures that are commonplace and relatively simple to adopt in other labs it is worth mentioning what barriers need to be overcome in order to facilitate ‘traditional’ rodent researchers adopting such a model and species.

 Specific comments:

 Authors state that a proportion of ATV-treated embryos did not develop ICH and therefore were used a controls – what proportion was unsuccessful?  Can the authors suggest why not all were successful? Did this result in a group of animals forming a control group which was treated separately to another control group (i.e. the untreated siblings)?

 Not clear to me (although I am a rodent researcher!) how a brief exposure to anaesthesia at 72hpf would remove locomotor function bias or was necessary? Couldn’t a baseline measurement be taken and data compared to this? The authors states that the exposure to this brief anaesthesia was at 72hpf but also state functional analyses were done at 72, 96 and 120 hpf – did the first time point occur coincidentally with the brief anaesthesia?

 Authors have made available all underlying source data.

**Are a suitable application and appropriate end-users identified?**

 Yes

**If applicable, is the statistical analysis and its interpretation appropriate?**

 Yes

**Are the 3Rs implications of the work described accurately?**

 Yes

Is the rationale for developing the new method (or application) clearly explained?

Yes

Is the description of the method technically sound?

Yes

Are the conclusions about the method and its performance adequately supported by the findings presented in the article?

Yes

If any results are presented, are all the source data underlying the results available to ensure full reproducibility?

Yes

Are sufficient details provided to allow replication of the method development and its use by others?

Yes

Reviewer Expertise:

Referee suggested by the NC3Rs for their scientific expertise and experience in assessing 3Rs impact.

I confirm that I have read this submission and believe that I have an appropriate level of expertise to confirm that it is of an acceptable scientific standard.

# Response

**Published:** 2018-11-02

**Authors:** siobhan crilly

**Affiliations:** The University of Manchester, UK

Dear Prof Gibson,

Many thanks indeed for reviewing our manuscript and your helpful comments. Please see below for responses to your specific questions.

***1. Authors state that a proportion of ATV-treated embryos did not develop ICH and therefore were used as controls – what proportion was unsuccessful?  Can the authors suggest why not all were successful? Did this result in a group of animals forming a control group which was treated separately to another control group (i.e. the untreated siblings)?***

The ATV treated non-haemorrhaged group formed the ICH- control group for the ATV model. As you suggest, this group was separate from the untreated control group, and our subsequent analyses compared all 3 groups (i.e. untreated, ICH- and ICH+ groups), as presented in Figure 2B and 3C. The proportion of ICH- controls varied with ATV dose, as presented in Figure 1B. For our cell death and motility assays, we used an ATV dosage of 1.0µg/ml, which resulted in approximately 20% of non-haemorrhaged animals, which we used as our ICH- group. We currently cannot explain why a proportion of ATV-treated larvae do not develop ICH. However as zebrafish are a relatively outbred model species, we hypothesise that a genetic component may explain increased neuroendothelial stability in some animals.

***2. Not clear to me (although I am a rodent researcher!) how a brief exposure to anaesthesia at 72hpf would remove locomotor function bias or was necessary? Couldn’t a baseline measurement be taken and data compared to this? The authors states that the exposure to this brief anaesthesia was at 72hpf but also state functional analyses were done at 72, 96 and 120 hpf – did the first time point occur coincidentally with the brief anaesthesia?***

Following fertilisation, we routinely plate n=50-100 embryos per petri dish. At 72hpf, natural variation in swimming behaviour/ability exists between individual larvae. For allocation to the locomotion assay, larvae are transferred from the petri dish into a 24 well plate using a pipette. To avoid always ‘catching’ the weakest/slowest swimmers, we briefly anaesthetise the animals so that larvae are randomly allocated to the assay. These fish were segregated at 72 hpf for repeat assays at 96 and 120 hpf and so there was no need to re-anaesthetise the animals for these later timepoints.

Kind regards

Siobhan Crilly & Paul Kasher
